# Dynamical ensemble of the active state and transition state mimic for the RNA-cleaving 8–17 DNAzyme in solution

**DOI:** 10.1093/nar/gkz773

**Published:** 2019-09-12

**Authors:** Şölen Ekesan, Darrin M York

**Affiliations:** Laboratory for Biomolecular Simulation Research, Institute for Quantitative Biomedicine, and Department of Chemistry and Chemical Biology, Rutgers University, Piscataway, NJ 08854, USA

## Abstract

We perform molecular dynamics simulations, based on recent crystallographic data, on the 8–17 DNAzyme at four states along the reaction pathway to determine the dynamical ensemble for the active state and transition state mimic in solution. A striking finding is the diverse roles played by Na^+^ and Pb^2+^ ions in the electrostatically strained active site that impact all four fundamental catalytic strategies, and share commonality with some features recently inferred for naturally occurring hammerhead and pistol ribozymes. The active site Pb^2+^ ion helps to stabilize in-line nucleophilic attack, provides direct electrostatic transition state stabilization, and facilitates leaving group departure. A conserved guanine residue is positioned to act as the general base, and is assisted by a bridging Na^+^ ion that tunes the p*K*_a_ and facilitates in-line fitness. The present work provides insight into how DNA molecules are able to solve the RNA-cleavage problem, and establishes functional relationships between the mechanism of these engineered DNA enzymes with their naturally evolved RNA counterparts. This adds valuable information to our growing body of knowledge on general mechanisms of phosphoryl transfer reactions catalyzed by RNA, proteins and DNA.

## INTRODUCTION

DNAzymes are DNA oligonucleotides capable of catalyzing variety of chemical reactions (1). Although not observed naturally, DNAzymes are of fundamental interest due to their ability to act as catalysts despite having a limited repertoire of functional groups and only modest structural diversity. Their greater stability, more ordered structure and absence in biology have practical advantages as biomedical tools and therapeutic agents for gene silencing (2) and virus control (3). Additionally, their ease of synthesis and identification make them particularly attractive for biotechnology applications (4) and some have been developed into sensors and imaging agents (5,6) for metal ions and bacteria. Hence, a general understanding of the mechanisms whereby DNA can act as an enzyme is of stand-alone significance.

A particularly important biological reaction is the sequence-specific RNA strand cleavage (7). RNA strand cleavage by 2′-*O*-transphosphorylation is universal in biology (8) and of fundamental importance to medicine (9). In biology, catalysis of this reaction frequently occurs through a general (or specific) acid/base catalytic mechanism. A base activates the 2′-OH group of the RNA substrate by deprotonation (either in a pre-equilibrium step, or concerted with nucleophilic attack). In the case of an associative mechanism, the activated nucleophile makes an in-line attack on the adjacent scissile phosphate, which then proceeds through a pentavalent phosphorane transition state to form 2′,3′-cyclic phosphate and 5′-OH cleavage products. In a subsequent step, the cyclic phosphate becomes hydrolyzed to generate a 3′ phosphate.

The 2′-*O*-transphosphorylation reaction is catalyzed in biological systems by protein ribonucleases such as RNase A (10) and the RNA ribonuclease RNase P (11), as well as small self-cleaving ribozymes (12–14) of which there are currently nine known classes. Much experimental and theoretical work has been directed at elucidating the detailed catalytic mechanisms of these natural biological enzymes (7). Artificial nucleic acid enzymes (15) have also been engineered to catalyze sequence-specific RNA cleavage, including the leadzyme RNA (16) and 8–17 DNAzyme (17–19). However, the mechanisms of these designed systems are not yet well understood, and it remains an open question as to the degree to which they may share common mechanistic features with their naturally evolved counterparts. The goal of gaining a predictive understanding of the catalytic mechanisms of RNA cleavage reactions exhibited by both natural and engineered biological molecules will enable general principles to emerge. These principles may ultimately be transferable outside the biomolecular enzyme scope and applied to guide the design of new technology built from synthetic systems, such as xeno nucleic acids (20) and Hachimoji DNA and RNA (21), which have great promise for new biotechnological applications (22).

An archetype RNA-cleaving DNAzyme that has been of great interest as a model catalytic DNA is the 8–17 DNAzyme (17–19). The 8–17 DNAzyme (8–17dz) active site contains a conserved guanine residue (G13) that functional studies suggest might act as a general base (23,24), similar to that of several endonucleolytic RNA enzymes. At near-physiological monovalent salt concentrations, 8–17dz requires divalent metal ions for activity. While 8–17dz is active with variety of metal ions, the rate of the reaction is sensitive to the identity of the metal, with Pb^2+^ being by far the most active metal (25). The current hypothesis is that the Pb^2+^ mechanism may be distinct from that of other divalent metals (e.g. Zn^2+^ and Mg^2+^), which FRET studies suggest requires further folding events to occur to achieve full activity (26) followed by an unfolding event that is prerequisite to release of the cleaved product (27). Further it has been shown that different ionic conditions induce different levels of folding and structural changes such as Z-DNA formation (28). Very recently, the crystal structure of the 8–17 DNAzyme (23) has been solved with a partially-occupied Pb^2+^ ion bound at the active site, providing a critical structural foundation from which theoretical studies may develop detailed dynamical models able to provide predictive insight into mechanism.

**Figure 1.**
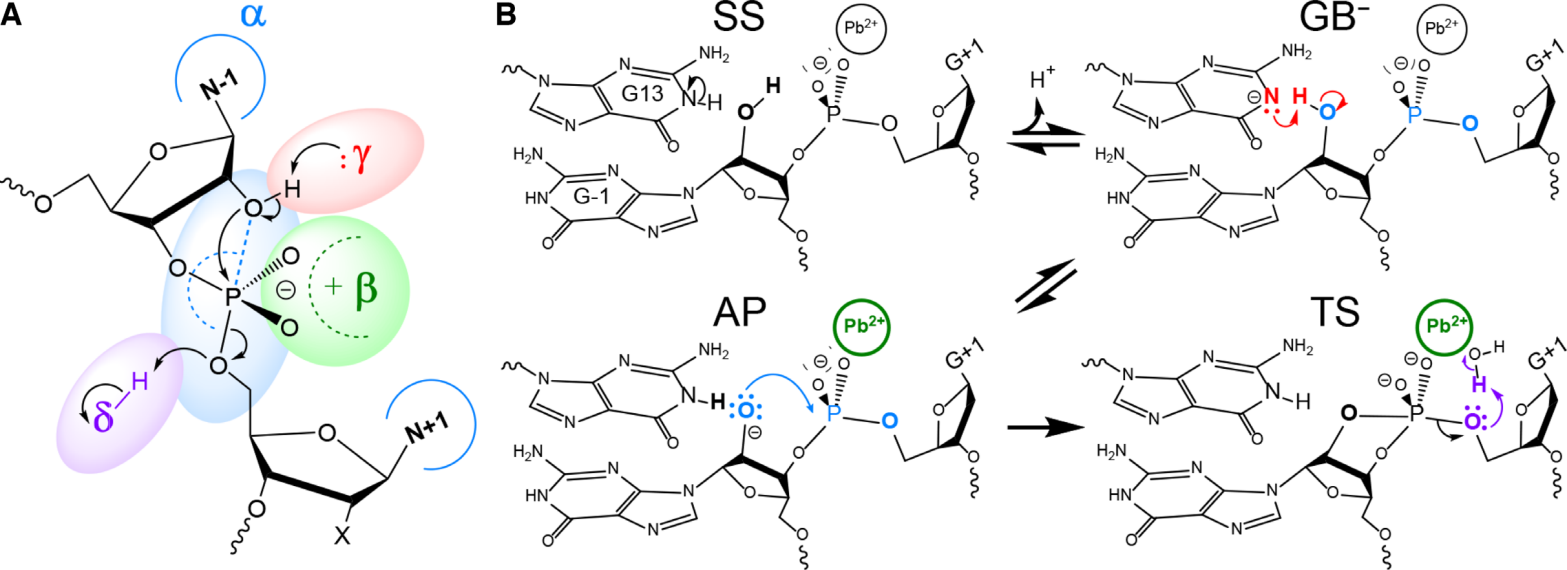
Catalytic strategies for 2′-*O*-transphosphorylation reaction leading to cleavage of the RNA backbone (7,29) highlighted on (**A**) schematic summary of the reaction based on the reactive state as a reference (i.e. GB^−^) and (B) along the reaction pathway of the proposed mechanism for the 8–17 DNAzyme. (A) γ (red), activation (deprotonation) of the 2′O nucleophile; α (blue), arrangement of the 2′O nucleophile, P (scissile phosphorus), and O5′ leaving group in an in-line attack geometry; β (green), stabilization (neutralization/protonation) of the negative charge accumulation on the nonbridging phosphoryl oxygens (NPOs); and δ (purple), stabilization (neutralization/protonation) of the accumulating negative charge on the O5′ leaving group. These general catalytic strategies (29) can be further formally decomposed into primary, secondary and tertiary contributions, in accord with a recently introduced ontology for more precise mechanistic discussions of RNA-cleavage reactions (7). Although this schematic is based on the general base deprotonated model to illustrate the fundamental catalytic strategies, these strategies can impact any state along the reaction pathway. (**B**) Standard state (SS), general base deprotonated state (GB^−^), activated precursor (AP) and transition state (TS) are shown. Key atoms participating in the catalytic strategies are emphasized in their corresponding color. Note: these states are modeled as idealized *stable states* to gain insight into how the DNAzyme environment provides catalytically relevant stabilization, and are not meant to imply a specific detailed chemical mechanism or reflect the true dynamical states along the reaction pathway.

Toward this end, we have performed molecular dynamics simulations of 8–17dz with an active site Pb^2+^ ion at four key states (designated SS, GB^−^, AP and TS) along the reaction pathway to explore the different fundamental catalytic strategies (7,29) used to enhance reactivity (Figure 1): stabilize in-line nucleophilic attack conformations (alpha catalysis), provide direct electrostatic transition state stabilization at the non-bridge phosphoryl oxygen positions (beta catalysis), activate (deprotonate) the nucleophile (gamma catalysis), and facilitate leaving group departure (delta catalysis). The present work builds from and complements pioneering crystallographic work (23) to provide new insight into how DNA molecules are able to solve the RNA-cleavage problem. These results add valuable information to our growing body of knowledge on general mechanisms of phosphoryl transfer reactions catalyzed by RNA (30), proteins (31) and DNA (32,33).

## MATERIALS AND METHODS

All simulations were carried out using AMBER18 (34,35), employing OL15 (36) DNA force field, with TIP4P/Ew (37) water model and corresponding monovalent ions from Joung and Cheatham (38) and divalent ion parameters from Li *et al.* (39). Simulations were performed under periodic boundary conditions at 300 K, using an 8 Å nonbond cutoff and electrostatics accounted for using the particle mesh Ewald (PME) method (40,41). Langevin thermostat with γ = 5 ps^−1^ and Berendsen isotropic barostat with τ = 1 ps are used for simulations in the NPT ensemble. Heavy atom time step of 2 fs is used along with SHAKE algorithm (42) for hydrogens. Frames are collected every 5 ps.

Simulations were conducted at four key states along the reaction pathway, as illustrated in Figure 1 and modeled as in previous work (43). These consist of the standard state (SS) with all residues in their standard (neutral) protonation state, activated general base (G13:N1 deprotonated) state (GB^−^), activated precursor (nucleophile G-1:O2′ deprotonated) state (AP) and a dianionic pentavalent phosphorane G–1/G+1 transition state mimic (TS) state were set up analogously. The standard state (SS) simulation system was setup departing from crystal structure of 8–17 DNAzyme with Pb^2+^-bound (PDB: 5XM8 (23)). 2′O nucleophile is modeled in at the G-1 position. The system was charge neutralized with Na^+^ counter ions, and then solvated by a truncated octahedron solvent box of 16 Å buffer distance. Bulk concentration of 0.14 M NaCl was then added, yielding a system with a total of 20k water molecules and ∼85k particles.

The system was solvent equilibrated in a multistage fashion through a cycle of minimization, NVT heating, and NPT equilibration where the constraint weight on the solute is approximately halved at each turn [50, 25, 10, 5, 2 kcal/(mol Å^2^)], yielding in a total of ∼50 ns solvent equilibration with constrained solute. Nonstandard state (GB^−^, AP and TS) simulations and/or simulations with different ion environments (i.e. Na^+^ only, without Pb^2+^) are derived from the standard state system with equilibrated solvent.

Each system was run for 100 ns and checked for stability (structure, energy, volume and temperature fluctuations) and convergence (root-mean-square positional deviations of structures). Convergence were achieved around 50 ns, after which 8 frames (16 for GB^−^ with Pb^2+^), 5 ns apart were extracted and used as starting points for production simulations. Simulations from each starting point were run for 150 ns (total of 1.2 μs), the first 25 ns of which were discarded as equilibration, yielding 1 μs trajectories for analysis (2 μs for GB^−^ with Pb^2+^). In this way, all analysis was made using 1–2 μs of equilibrated data from 8 independent simulations for each state. The use of such an ensemble approach has been demonstrated to lead to more efficient sampling of different free energy basins relative to longer single-run simulations (44,45). Further, convergence of plots has been estimated by considering statistical sampling from only a subset of simulations, and examining overlap distributions from the cumulative 1–2 μs of data derived from the 8 independent simulations.

Angles, distances and radial distribution functions were obtained using corresponding modules of cpptraj (46). Radial distributions were obtained using bin size of 0.05 Å. Catalytic strategy (Figure 1A) analysis plots are calculated and normalized in a strategy-specific manner. For each item an index is defined that measures the ‘fitness’ of each catalytic strategy based on established geometrical metrics (e.g. H-bond, ion-coordination, nucleophile attack distances, in-line angles, etc.) with thresholds that have been used in the recent literature (details given in the Supplementary Data). A color gradation scheme (0–1) is then used to reflect the degree of catalytic fitness through color intensity (i.e. white indicating absence of catalytic fitness). The definition of each catalytic strategy, along with its base color is shown in Figure 1.

## RESULTS AND DISCUSSION

In this section, we present and discuss results of multiple independent molecular dynamics simulations of 8–17dz, and use them to aid in the interpretation of recent experimental structural and functional data. Throughout the manuscript we place discussion in the context of four fundamental catalytic strategies (29) for RNA cleavage via 2′-*O*-transphosphorylation, designated α, β, γ and δ, illustrated in Figure 1A. These general catalytic strategies (except α) can be further formally decomposed into primary, secondary, and tertiary contributions as described in a recently presented ontology for discussion of mechanisms for RNA-cleaving enzymes (7). Briefly, primary contributions are those that directly alter the identity of ‘primary atoms’ directly involved with the chemical space of bonds associated with a particular catalytic strategy, whereas secondary contributions are caused by a change in the electronic environment resulting from changes in the identity of non-primary atoms. Tertiary contributions are those that arise from modification of the structural scaffold or hydrogen bond network that organizes the enzyme active site.

In order to connect our simulation results with each catalytic strategy, we have performed simulations at four key states along the reaction pathway, which are illustrated in Figure 1B. The standard state (SS) has all residues in their standard protonation state, the activated general base (GB^−^) state has G13 deprotonated at the N1 position so as to be able to deprotonate the nucleophile (G-1:O2′) to form the activated precursor (AP) state, and then goes on to make an attack on the adjacent scissile phosphorus to form the dianionic pentavalent transition state (TS) mimic.

Before proceeding, we would like to clarify some terminology and concepts regarding acid/base catalysis (47,48), and relate them to the simulations in the current study. In the present work, we examine models that mimic states along the presumed reaction pathway illustrated in Figure 1 using classical molecular dynamics. In doing so, these states are modeled as *stable states* in order to gain insight into how the DNAzyme environment can provide stabilization relevant for catalysis. It should be emphasized that in the catalytic mechanism itself, some of these states will be transient species along the reaction pathway, and not necessarily stable. For example, if the ‘activated precursor’ (AP) state illustrated in the Figure 1B was a stable state where nucleophile activation was achieved in a proton transfer involving rapid equilibrium prior to formation of the rate controlling transition state, then this would imply a specific base catalysis mechanism. Further, note that our ‘transition state’ (TS) mimic model is an idealized dianionic pentavalent phosphorane species, with no partial proton transfer from the general acid. In the actual catalytic mechanism, the reaction could conceivably proceed through a protonated phosphorane intermediate, or a rate-controlling transition state that involves partial proton transfer to the leaving group.

In the results and discussion below, we will refer to the states illustrated in Figure 1B, and also make reference to residues such as G13 as the presumed ‘general base’. However, we emphasize that precise statements about the specific chemical steps of the catalytic mechanism will require quantum mechanical simulations (49,50) and possibly additional experimental measurements (51–55) that are discussed briefly in the Supplementary Data.

In the sections that follow, we discuss general features of the 8–17dz and its active site, describe the requirements for formation of the active state in solution, and characterize the specific mechanisms 8–17dz uses to support catalytic strategies at different points along the reaction pathway. The paper concludes by discussing the diverse role of metal ions in 8–17dz catalysis, and placing these results in the broader context of similar naturally occurring RNA enzymes.

### Overall fold and active site architecture

**Figure 2.**
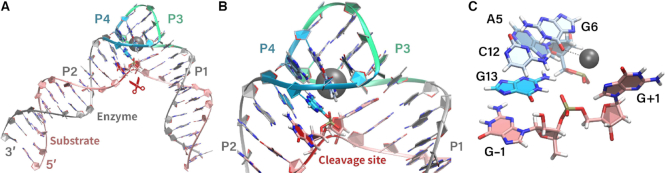
Crystal structure of the Pb^2+^ bound 8–17 DNAzyme (PDB ID: 5XM8 (23)) depicting (**A**) the overall fold, (**B**) catalytic core and (**C**) architecture of the active site with residues participating in catalysis. The four stems of the enzyme are colored as: gray for substrate binding P1 and P2 stems, green for P3 stem containing canonical Watson–Crick (WC) pairing, and blue for the P4 stem with the catalytic conserved residues. The substrate strand is shown in pink (with the cleavage site highlighted in red in panels A and B). The proposed general base G13 is shown in blue and the remaining conserved residues are shown in light blue. The coordination of Pb^2+^ (dark gray) to G6:O6 is shown in black dashed line.

The recent crystal structure of 8–17dz (23) provides key insight into the overall fold and architecture of the active site, and serves as a critical departure point for theoretical prediction of the dynamical ensemble in solution at different stages along the reaction pathway. The overall fold of 8–17dz is ‘V’-shaped, with P1 and P2 helical stems forming extended arms responsible for recognition of the substrate via canonical base pairing (Figure 2). The catalytic core consists of 15 nucleotides that form a compact twisted pseudoknot containing two short helices (P3 and P4), oriented perpendicularly to one another. P4 contains four of the five highly conserved nucleotides (A5, G6, C12, and G13, in addition to C7) (56,57); G6 and C12 form a canonical WC C=G pair, and A5 and G13 a non-canonical A
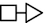
G pair (58,59). G13 and G6, are proposed to be the general base (23,24) and metal ion binding site (23), respectively.

Interactions among key residues cause a GG-kink (23) to occur at the substrate cleavage site, causing G –1 and G+1 to be splayed apart. Note: we refer to the *N*–1 residue as G in the current work, despite that in some of the DNAzyme literature it is designated as rG (owing to the fact that it is the only RNA residue). This splaying of the substrate nucleobases flanking the scissile phosphate stabilizes the nucleophile to be in an in-line attack configuration, and is commonly seen in naturally occurring endonucleolytic ribozymes (7,60). The 8–17dz fold, facilitated by interactions of conserved and key residues (discussed in further detail in Supplementary Data and illustrated in Supplementary Figure S1), creates an architecture and electrostatic environment for the active site that enables key catalytic strategies to be brought to bear to enhance activity.

### 8–17 DNAzyme has engineered an electrostatically strained active site that recruits metal ions to assist in catalysis

It is an interesting chemical question as to how nucleic acids, having available only a limited diversity of fairly unreactive building blocks, can be designed to act as highly efficient catalysts. One strategy that has been observed in several small RNA enzymes, is to arrange charged residues in the 3D structure so as to create an electrostatic cation recruiting pocket to attract metal ions that can participate in catalysis (61–64). These metal ion binding sites often occur at junctions where negatively charged helices come together, which is part of the reason why the active sites are also found at these locations.

**Figure 3.**
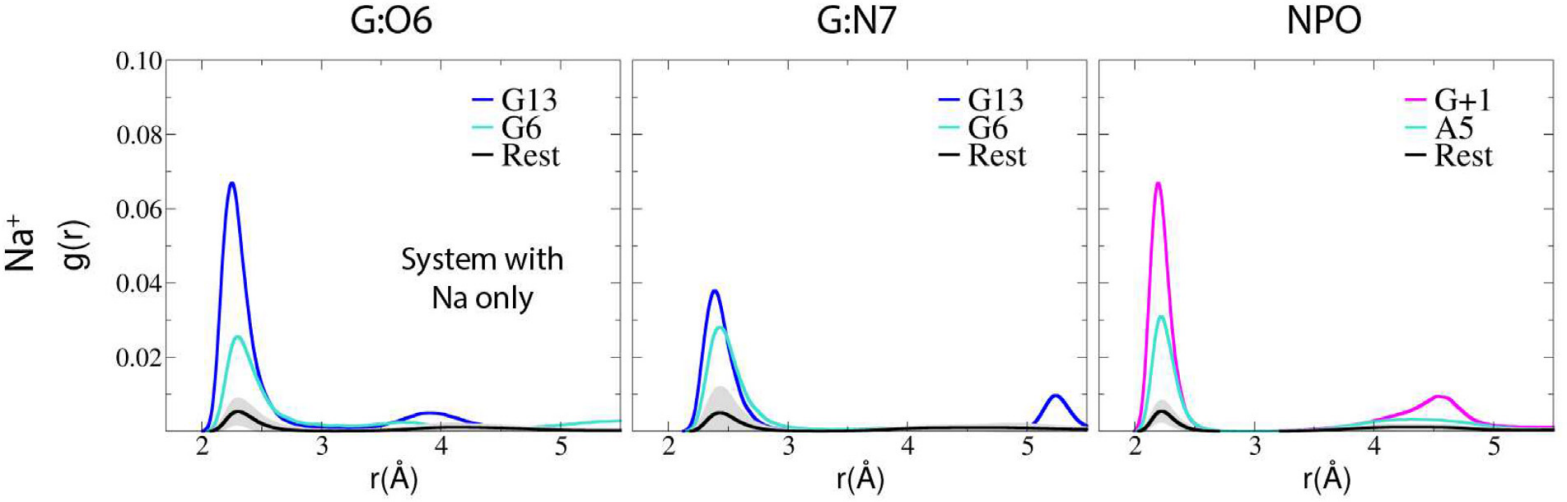
Radial distribution of Na^+^ ions around O6 and N7 of all guanines and all nonbridging phosphoryl oxygens (NPOs) obtained from solution simulations of the general base deprotonated (GB^−^) 8–17 DNAzyme system with only Na^+^ ions, referred to in text as ‘Na only’. The two highest peaks are explicitly shown and are excluded from the averages. Average of all remaining residues and their standard deviations are shown respectively by the black curve and the gray shaded area.

The 8–17 DNAzyme is no exception, with the catalytic core formed at the pseudoknot where all four helices come together. MD simulations in 140 mM Na^+^ ions (and no divalent metal ions) indicate that an electrostatic binding pocket is formed by the pro-*R*_P_ NPO of the scissile phosphate (G+1), the Hoogsteen edge of G6 and the pro-*R*_P_ NPO of A5. Monovalent ions can bind to nucleic acids both territorially (65,66) or in some cases (such as in G-quadruplexes and some enzymes) site specifically (66). In the simulations, Na^+^ ions are observed to predominantly territorially bind, and in instances where they make inner-sphere contact with nucleobase functional groups such as the Hoogsteen edge of guanine residues, exchange is observed on the tens of ps time scale. Evidence of Na^+^ exchange is also supported by the radial distribution functions (RDFs) in that they have finite (non-zero) probability between the first and second peaks in Figure 3. Radial distribution functions of Na^+^ ions around these positions indicate that cations preferentially are attracted to this binding pocket with respect to other G residues and phosphate NPO positions (Figure 3 and Supplementary Figure S2). This is consistent with crystallographic data that suggest that a Pb^2+^ ion binds with partial occupancy to the Hoogsteen edge of G6 (23), as well as phosphorothioate substitution results that indicate the pro-*R*_P_ of the scissile phosphate is a binding site for inner-sphere coordination of a catalytic divalent metal ion (67). Additionally, the active site attracts cationic charge from solvent at the solvent-exposed Hoogsteen edges of bases G13 and G6 (Figure 2). As will be discussed below, the electrostatic engineering of the 8–17dz active site enables binding of both monovalent and divalent metal ions that can play an organizational role as well as directly participate in the chemical steps of catalysis.

### Identifying the requirements for formation of the active state, and characterizing its probability (abundance) in solution

The ‘active state’ of 8–17dz is the state that is catalytically competent to carry out the chemical steps of catalysis. This implies that the DNAzyme has the correct structure, protonation state and metal ion binding mode required for activity. In addition to having the overall correct fold, the active state structure must also have the correct arrangement of residues in the active site. First of all the general base needs to be in the protonation state that would allow it to initiate the reaction. This is denoted as the general base deprotonated state (GB^−^ illustrated in Figure 1B). Further, the 2′-OH nucleophile must be in hydrogen bonding position to enable activation (γ catalysis) by the general base (G13:N1). Once activated, the reactive nucleophile should be aligned with the P atom of the scissile phosphate and O5′ leaving group in a near-linear (typically > 140°) in-line geometry as well as productive proximity to the scissile phosphate (α catalysis). This geometry facilitates attack on the phosphorus that typically proceeds through a dianionic trigonal bipyramidal pentacovalent transition state in accord with so-called Westheimer’s rules (68) for phosphate ester reactivity.

However, unlike many protein enzymes, it is often the case that the active state of a nucleic acid enzyme is rare (i.e. has low abundance even at optimal conditions) and is sensitive to both pH and ionic conditions. Crystallographic structures of nucleic acid enzymes are invaluable in providing insight into the overall fold, but most often do not represent the catalytically active states in solution. Nonetheless, these structures serve as a critical departure point for rigorous computational modeling studies aimed at predicting the active state conformational ensemble in solution. This is challenging due to the need to explore local conformational changes, and alternative protonation states and metal ion binding modes that are tightly coupled.

Here, we have performed microsecond MD simulations of 8–17dz with the general base guanine (G13) deprotonated at the N1 position in order to uncover the conformational and metal ion binding requirements for formation of the catalytically active state in solution. Separate simulations were performed both in the presence and absence of a Pb^2+^ ion observed crystallographically with partial occupancy in one of the two asymmetric units, and both of these simulations were carried out in a background of neutralizing Na^+^ and 140 mM NaCl. A total of 16 and 8 independent 150 ns simulations were carried out for systems with Pb^2+^ and without Pb^2+^, respectively. These simulations were monitored for active state population, where the ‘active state’ was defined as being positioned for nucleophilic activation (γ catalysis) through formation of a hydrogen bond between 2′-OH and G13:N1, and being poised for nucleophilic attack (α catalysis) with productive in-line angle (θ_inl_ ≥ 140°) and O2′-P distance (≤3.5 Å). This definition is consistent with the requirements for the chemical steps of the reaction, as has been demonstrated in other recent work on RNA enzymes (50).

**Figure 4.**
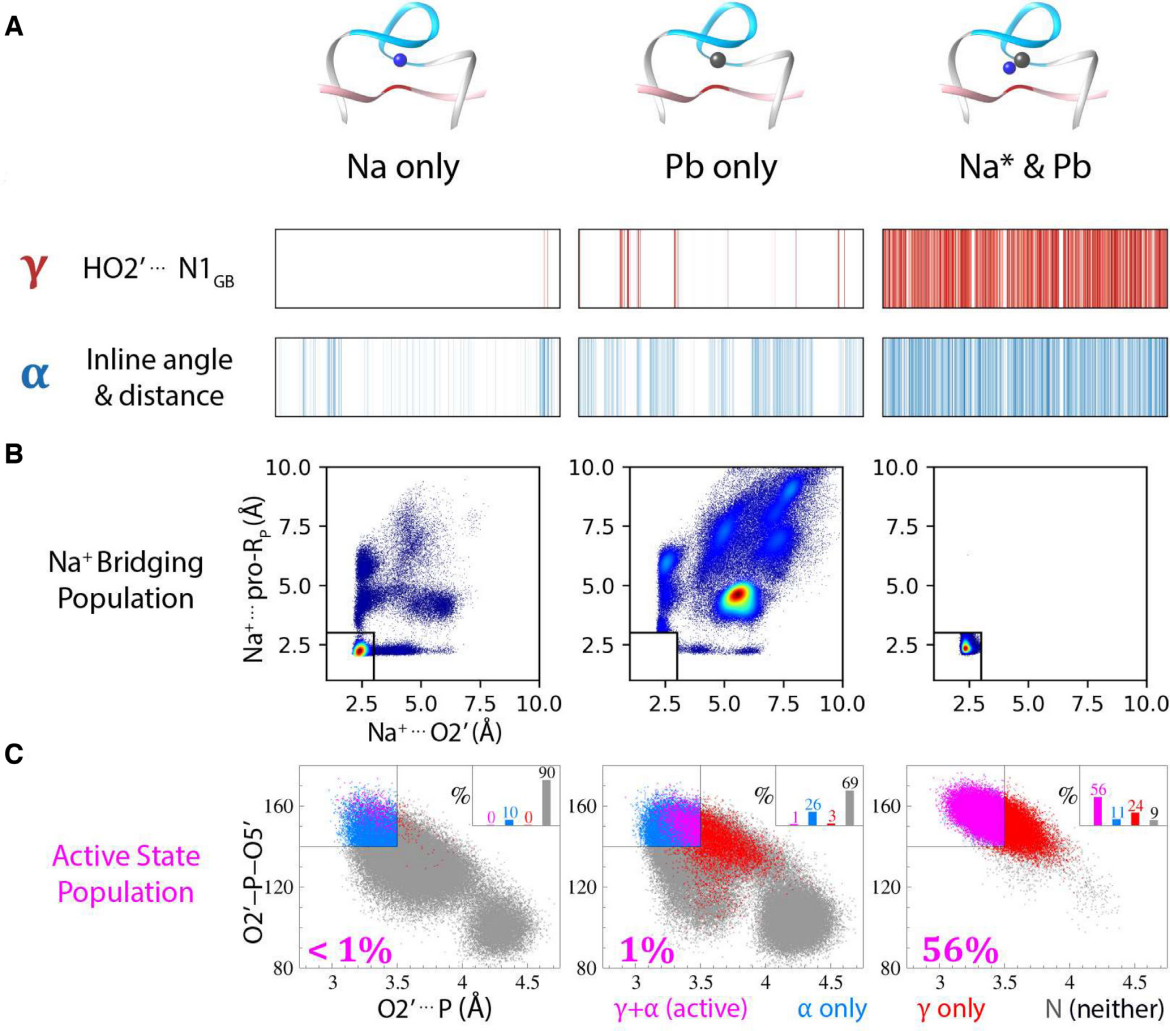
Analysis of general base deprotonated state (GB^−^) trajectories in search of the active state in solution. Trajectories for systems with Pb^2+^, and without Pb^2+^ ion (designated ‘Na only’) are shown. Trajectories for system with Pb^2+^ ion are shown as two clusters based on metal ions present at the active site; with only Pb^2+^ (designated ‘Pb only’) and with Na^+^ ion in bridging position between O2′ and pro-*R*_P_ (designated ‘Na* and Pb’). ‘Na only’, ‘Pb only’ and ‘Na* and Pb’ results correspond to 1 μs, 490 ns and 1.5 μs of trajectories, respectively. (**A**) Depiction of frames with active site positioned for nucleophile activation (γ, red) and in-line attack (α, blue) along the simulation trajectory. Frames matching the criteria for in-line attack (α: O2′⋅⋅⋅P ≤ 3.5 Å and ∠O2′-P-O5′ ≥ 140°), and nucleophile activation by general base (γ: HO2′⋅⋅⋅G13:N1 ≤ 2.2 Å) are shown in their corresponding colors, with darker tones indicating better catalytic fitness. (**B**) Na^+^ ion population around 2′O nucleophile and pro-*R*_P_ oxygens with densities shown as increasing from blue to red. (**C**) Active state population shown as percentage of the overall frames in the category. Active state is defined as active site structure in position for both in-line attack (α) and nucleophile activation (γ). Points on the scatter plot are colored such that frames matching the criteria for only α or only γ are shown in blue and red, respectively. Frames that match criteria for both α and γ (i.e. active) are shown in magenta, and frames that do not match either criteria are shown in gray.

Analysis of the cumulative simulation results (Figure 4) suggest that in the absence of Pb^2+^ in the active site (i.e. ‘Na only’), an active state is observed <1% of the time. Alternatively, for the simulations with Pb^2+^, the active state was observed about 15% of the time. Clustering analysis of the data (Supplementary Figure S3 and Supplementary Table S1) indicated that when the active state was observed, it was almost always accompanied by a Na^+^ ion that bridged the O2′ and the pro-*R*_P_ of the scissile phosphate. It should be pointed out that multiple independent simulations sampled both ‘Pb only’ and ‘Na* and Pb’ states, and the same correlation with inactive and active states was observed in each, lending support that the conclusions from our ensemble approach are not due to convergence artifacts (see Materials and Methods section).

In order to ascertain the degree to which this was a requirement for formation of the active state, the simulation data was re-clustered (Supplementary Figure S4) into two sets, one with only Pb^2+^ in the active site (i.e. without a bridging Na^+^), designated ‘Pb only’, and another where Na^+^ ion is in the bridging position, designated ‘Na* and Pb’ (where ‘Na*’ is used to indicate the presence of the bridging Na^+^ in the active site). The data for these simulations (Figure 4) indicates that, in the absence of binding of a Na^+^ ion to the 2′-OH, formation of the active state occurs around 1% of the time, whereas when a Na^+^ ion bridges the 2′-OH and the pro-*R*_P_ oxygen, formation of the active state occurs around 56% of the time.

Taken together, these results suggest that Pb^2+^ ion binding in the active site is a necessary, but not sufficient condition for formation of the active state (with high abundance). Rather, the additional binding of a Na^+^ ion in a bridging position between the 2′-OH nucleophile and the pro-*R*_P_ position of the scissile phosphate is requisite to enhance the formation of the active state in solution.

### Simulations at key stages along the reaction pathway provide insight into the different modes of catalysis

**Figure 5.**
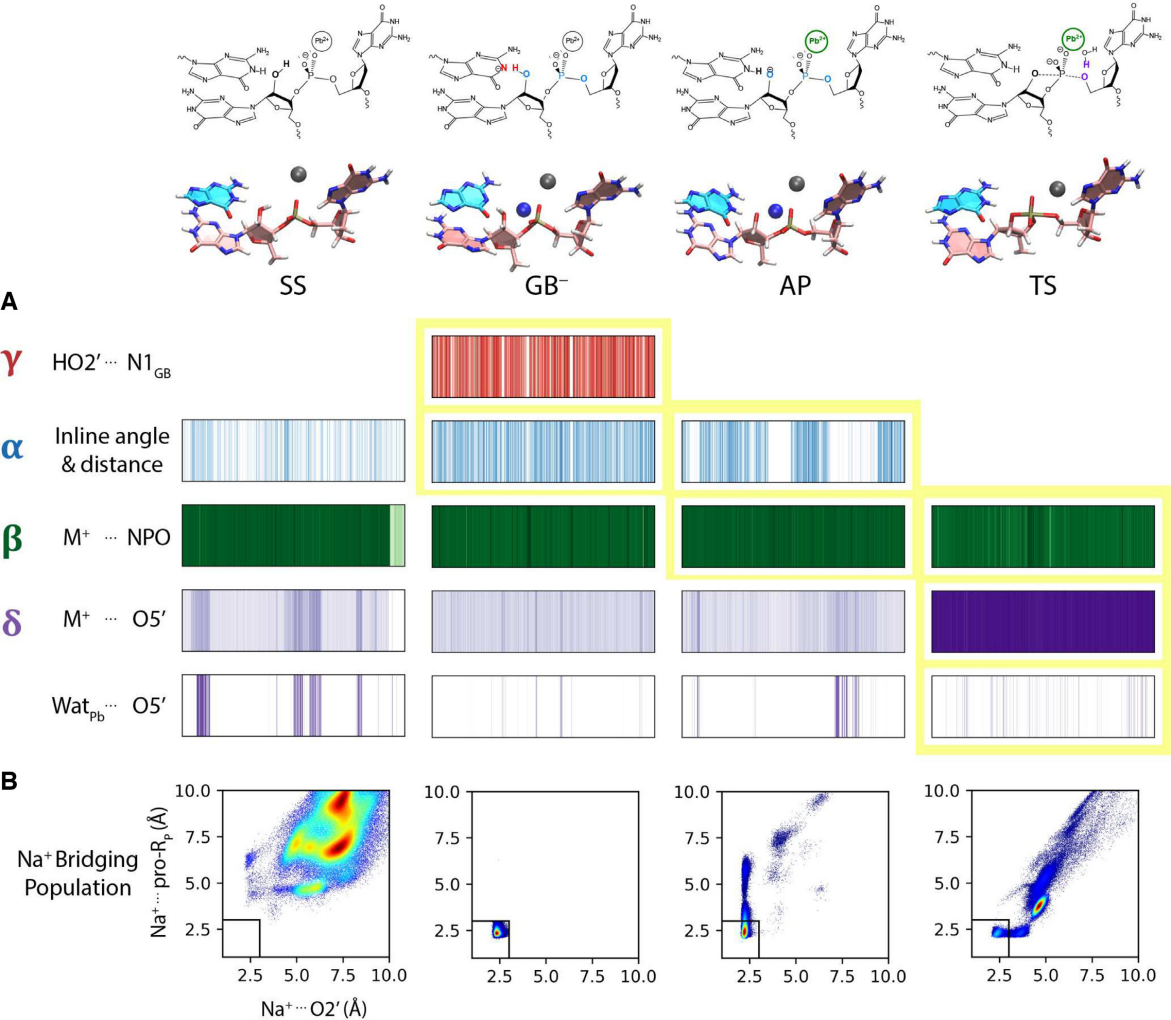
Catalytic strategies and Na^+^ ion positioning in the active site along the reaction pathway from simulations with Pb^2+^. Four studied states; standard state (SS), general base deprotonated state (GB^−^), activated precursor state (AP), and transition state mimic (TS) are reported. SS, AP and TS trajectories correspond to 1 μs whereas GB^−^ result is on the set of frames in the cluster with Na^+^ bridging between O2′ and pro-*R*_P_ (‘Na* and Pb’, 490 ns). (**A**) Depiction of frames with active site positioned for nucleophile activation (γ, red), in-line attack (α, blue), charge stabilization of NPOs (β, green), and stabilization of the leaving group (δ, purple) along the trajectory. Frames matching the criteria for each strategy are shown in their corresponding colors, with darker tones indicating better catalytic fitness. α and γ criteria are the same as Figure 4. β criteria is based on direct coordination distance of a metal ion (Pb^2+^ and/or Na^+^) to the NPOs. Two sets of analyses are shown for δ; metal ion coordination to the leaving group, and Pb^2+^ coordinated water hydrogen bonding to the leaving group. For metal ion coordination light and dark colors illustrate second and first solvation shell coordination, respectively. Criteria for Pb^2+^ coordinated water is based on the hydrogen bond distance to the leaving group O5′. Strategies are highlighted in yellow to depict the state in which their presence is essential for catalysis. (**B**) Na^+^ ion population around 2′O nucleophile and pro-*R*_P_ oxygens with densities shown as increasing from blue to red.

**Figure 6.**
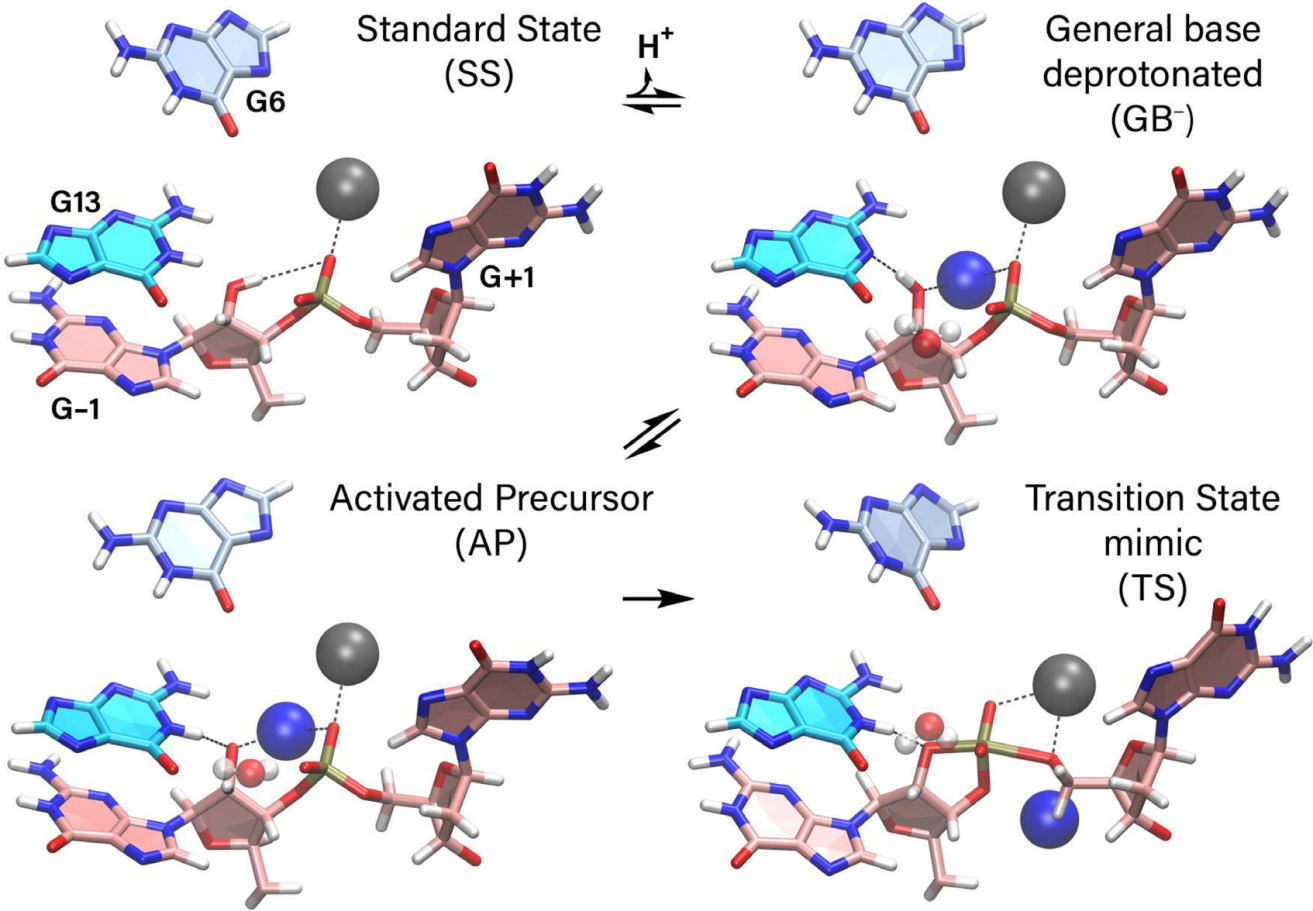
Solution structures of 8–17 DNAzyme from simulations of states along the reaction pathway with Pb^2+^. Structures shown correspond to averages over trajectories. Cleavage site residues G–1 and G+1 on substrate strand are shown in pink, general base guanine G13 is in blue, and the conserved binding pocket residue G6 in light blue. Solvent molecules are shown when present in catalytically relevant positions. Metal ions are shown in van der Waals representation, Na^+^ in blue and Pb^2+^ in dark gray; and water molecules in CPK representation rendered transparent (percent presence of solvent bridging G13:O6 and pro-*S*_P_ NPO are listed in Supplementary Table S2). Interactions within hydrogen bond or direct coordination distances are illustrated with black dashed lines.

With the requirements for formation of the active state identified, we consider sequentially several stages along the reaction pathway illustrated in Figure 1. As discussed previously, the ‘SS’ (standard state) is pre-reactive state that is expected to be the most populated state at neutral pH and active ionic conditions (e.g. 140 mM NaCl and 0.5 mM PbCl_2_ as was in the crystal buffer (23)). The ‘GB^−^’ active state, on the other hand is the reactive state that is competent to initiate the cleavage reaction, the first step of which is activation (deprotonation) of the 2′-OH nucleophile. Whereas the SS has the general base (G13) in its standard (neutral) protonation state at pH 7, the active state ‘GB^−^’ requires the general base to be deprotonated at the N1 position. The activated precursor ‘AP’, is the state that results after the general base abstracts the proton from the 2′-OH nucleophile, which is now activated for nucleophilic attack. The transition state (mimic), designated ‘TS’, is then achieved by the activated nucleophile that makes an attack on the scissile phosphate to form a dianionic trigonal bipyramidal transition state or metastable intermediate. The term ‘mimic’ is used here, as we are not using quantum electronic structure methods to compute a true transient transition state, but rather have created a stable molecular mechanical model that has the geometry and partial charge distribution of a true transition state, but unlike a true TS, is stable as the pentavalent species (similar to an experimental vanadate transition state mimic, but a more accurate representative of a phosphoryl transfer TS). Figure 5 summarizes results from simulations of each of these states in the context of the four fundamental catalytic strategies (Figure 1). Representative structures for each of these states are illustrated in Figure 6.

In none of the standard state (SS) simulations does the Pb^2+^ ion stay stably bound in the crystallographic binding mode at G6:O6 where it is observed with partial occupancy for one of the asymmetric units (23). The coordination environment of the Pb^2+^ ion undergoes small changes, but Pb^2+^ remains in the active site in all simulations. This result, however, does not definitively exclude the crystallographic binding mode as one that can also exist in solution with partial occupancy and possibly have catalytic activity. It has been estimated that Pb^2+^ ions can bind stably to the Hoogsteen edge of guanine residues (69). Nonetheless, our simulations suggest that the electrostatically preferred binding mode in solution is at the pro-*R*_P_ position, and this is consistent with reported reference to phosphorothioate modification results as functionally important for Mg^2+^ ions (67). Overall, the SS simulation is observed to visit states that have in-line fitness, as would be required for nucleophilic attack, but these configurations are correlated with hydrogen bonding between the 2′-OH nucleophile and the NPOs of the scissile phosphate. While these hydrogen bonding interactions help to stabilize in-line fitness, they are non-productive in the sense that the 2′-OH must ultimately form a hydrogen bond with the G13:N1 position in order for nucleophile activation to occur. The presence of the H1 proton in the SS simulation prevents the G13:N1 heteroatom from receiving this critical hydrogen bond.

In the simulations of the GB^−^ state, the presence of a bridging Na^+^ ion recruited from solution, as described above, stabilizes both in-line fitness and proper position of the deprotonated general base G13:N1 to activate the nucleophile. The Na^+^ ion forms a critical bridge between the O2′ and the pro-*R*_P_ NPO, substituting even more effectively for the role of the hydrogen bond between the same positions that stabilized in-line fitness in the SS simulations. As will be discussed in more detail below, the Na^+^ ion in this position also acts to tune the p*K*_a_ of the nucleophile so as to increase its acidity and facilitate abstraction of the proton by the general base. This tuning of the nucleophile p*K*_a_ is a form of secondary γ catalysis (i.e. inducing a change in the electrostatic environment to facilitate the nucleophile activation) (7).

The simulations of the AP state describe the DNAzyme-substrate complex after nucleophile activation. Deprotonation of the nucleophile leads to a greater localized charge at the 2′O position that strengthens the interaction with Na^+^, and consequently preserves in-line fitness supported by the bridging Na^+^ binding mode. The AP state is a high-energy state that is not expected to be long-lived, and will either make an attack on the scissile phosphate or else revert back to its protonated (neutral) form. It is noteworthy that the Pb^2+^ binding mode at the pro-*R*_P_ NPO predicted by the simulations helps to facilitate the nucleophilic attack by partially neutralizing the negatively charged phosphate center, lowering the barrier for approach of the anionic nucleophile. The direct (inner-sphere) binding of the Pb^2+^ ion to the pro-*R*_P_ NPO is a form of primary β catalysis (7).

The simulations of the TS are perhaps the most relevant for 8–17dz catalysis. The TS represents a dianionic transition state, which requires electrostatic stabilization of the NPOs (β catalysis), in addition to stabilization of the accumulating charge of the leaving group (δ catalysis). The binding of the Pb^2+^ ion at the pro-*R*_P_ has a primary β catalytic effect as discussed above. However, in the TS simulation, the Pb^2+^ ion is observed to interact with the 5′O leaving group through direct (inner-sphere) coordination whereas the crystal structure depicts indirect (outer-sphere) coordination with a partially occupied Pb^2+^ ion making inner-sphere coordination to G6:O6, and a metal-bound water at a hydrogen bond distance with the 5′ leaving group. We tested this binding mode through enforcing crystallographic distances between the Pb^2+^ and G6:O6 or G+1:O5′ to maintain direct coordination to G6:O6 or outer-sphere coordination to G+1:O5′, respectively. In both cases, constrained simulations indicated at least one water molecule quickly filled the gap between Pb^2+^ and O5′ with an orientation to donate a hydrogen bond to O5′ (Supplementary Figure S5). However, as soon as the restraints were released, the Pb^2+^ ion fell back into a position directly coordinating the O5′.

Although our simulations cannot rule out the crystallographic binding mode as catalytically relevant, the direct coordination to pro-*R*_P_ binding mode observed in simulations is consistent with the referenced pro-*S*_P_-selective cleavage with Mg^2+^ ions (67). These results underscore the importance, when reconciling structural and functional data, to consider not only the pre-reactive or even the active state, but also states further along the reaction pathway, particularly the TS, that could affect activity (70).

### Binding mode of Pb^2+^ and Na^+^ ions along the reaction path

**Figure 7.**
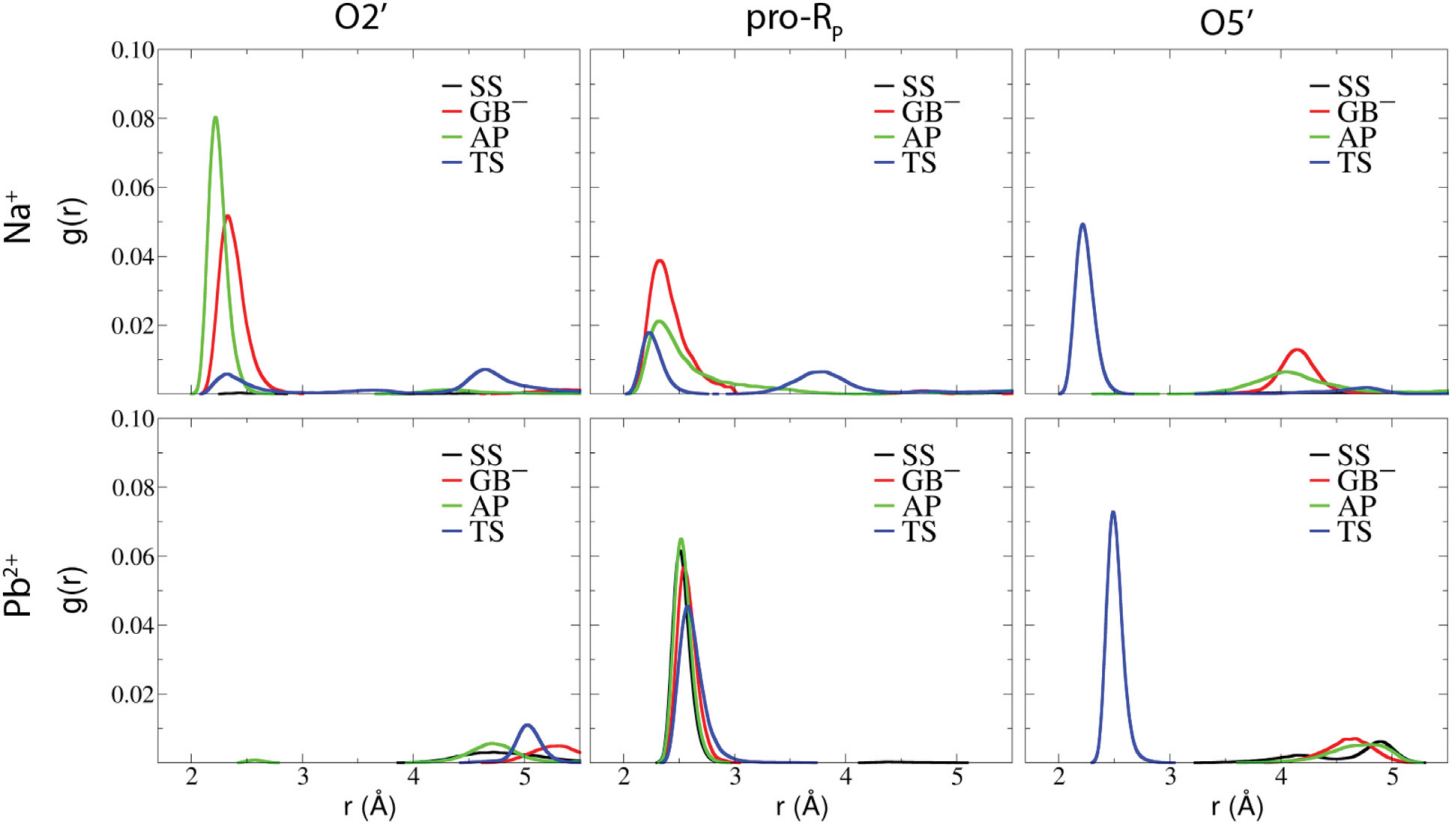
Binding modes of metal ions along the reaction pathway. Radial distribution, g(r), of Na^+^ and Pb^2+^ ions around nucleophile O2′, scissile phosphate pro-*R*_P_ and leaving group O5′ obtained from solution simulations of standard state (SS, black), general base deprotonated state (GB^−^, red), activated precursor state (AP, green), and transition state mimic (TS, blue) with Pb^2+^ consistent with data in Figure 5. SS (black) lines often invisible due to negligible peaks.

In this section we focus on the metal ion binding modes at different stages along the reaction pathway, and discuss the diverse roles they play in 8–17dz catalysis. As discussed previously, the 8–17dz active site is located at the junction of P1-P4 helices where it forms a compact pseudoknot structure, and has been designed to be highly electronegative so as to be able to recruit monovalent and divalent metal ions from solution. In particular, a Pb^2+^ ion plays a critical role in the chemical steps of catalysis, and this is supported by transient binding of a Na^+^ ion that bridges the 2′O nucleophile and pro-*R*_P_ of the scissile phosphate. Representative structures for these binding modes are illustrated in Figure 6. We now quantify the key interactions of these two ions using radial distribution functions around key atomic positions in order to illustrate how these metal ion binding modes evolve along the reaction pathway. RDFs for Na^+^ and Pb^2+^ ions around the nucleophile (O2′), scissile phosphate pro-*R*_P_ NPO (*R*_P_), and leaving group (O5′) positions are shown in Figure 7 for the SS, AP, GB^−^ and TS states.

The Pb^2+^ ion binds stably at the pro-*R*_P_ position in all of the simulations. In this position, it has negligible interaction with the 2′O nucleophile, with minor exception that there is a slight interaction around 5 Å (indicated by the small peak at this distance value) that occurs in the TS simulation where the O2′ has formed a partial covalent bond and is in closer proximity to the scissile phosphorus. A striking feature of the Pb^2+^ RDFs is that the appearance in the TS simulation of a pronounced peak around 2.5 Å from the O5′ leaving group. This suggests that there are strong interactions between the Pb^2+^ ion and the leaving group in the TS that are not apparent earlier along the reaction path.

The binding of Na^+^ in the active site is more varied and dynamic. Na^+^ ions are observed throughout the simulations to bind ‘territorially’ around the active site (65,66,71). In addition to this delocalized binding mode, in the GB^−^ and AP simulations, Na^+^ can bind in a bridging position where it makes inner-sphere contact with both the 2′O and the pro-*R*_P_ positions. In the GB^−^ simulation, strong inner-sphere Na^+^ coordination peaks occur around 2.4 Å for both these positions. In the AP simulation where the O2′ is deprotonated to form an oxyanion, the Na^+^ coordination peak with this position becomes more pronounced and shifts to shorter distance, and the interaction with the pro-*R*_P_ becomes slightly less than it is in the GB^−^ simulation. It is interesting to note that there is only minor interaction of Na^+^ ions with the O5′ position, the significant exception being the appearance of a high inner-sphere coordination peak around 2.2 Å in the TS simulation.

In addition to the bridging Na^+^ binding mode, it was observed that pronounced Na^+^ binding occurred at the Hoogsteen edge of the general base, and specifically around G13:O6. A striking result is that binding of Na^+^ ions at G13 and G6 that forms the functionally relevant active site binding pockets are by far the most prominent G positions throughout the 8–17dz (Figure 3). Both G13 and G6 are not involved with base pair interactions at their WC face, and have their electronegative Hoogsteen edges exposed to solvent. This Na^+^ binding mode for G13 is most pronounced in the GB^−^ state (Supplementary Figure S6), where the negative charge of the deprotonated guanine is delocalized between the N1 and O6 positions.

### Diverse role of metal ions in 8–17dz catalysis

#### Role of Pb^2+^ (divalent) metal ion

The Pb^2+^ ion helps to organize the active site, as indicated by comparison of simulation in the presence and absence of Pb^2+^ (Figure 4) that show Pb^2+^ enhances in-line fitness (α catalysis). In addition, Pb^2+^ binding at the pro-*R*_P_ position, together with Na^+^ binding (see below), facilitates productive hydrogen bonding with the G13:N1 heteroatom by elimination of non-productive hydrogen bonding to the NPO. This mode of catalysis was first suggested for the *glm*S ribozyme (72,73), where it was referred to as ‘gamma prime-prime’ (γ″), but in accord with a recently introduced ontology (7), is designated as a form of tertiary γ catalysis resulting from modification of the structural scaffold or hydrogen bond network (in this case the latter) that organizes the enzyme active site to support nucleophile activation. The Pb^2+^ ion primarily provides critical electrostatic stabilization of the dianionic transition state (β catalysis), and accumulating charge in the leaving group (δ catalysis). Stabilization of the leaving group in the simulations was observed primarily through inner-sphere coordination.

The model for Pb^2+^ ion in our simulations was designed to reproduce different experimental properties, including hydration free energies, coordination numbers and ion-oxygen radial distribution functions, appropriate for use with the PME method (40) and AMBER force field that uses Lennard–Jones parameters with Lorentz–Berthelot combining rules. Nonetheless, modeling metal ion interactions in biomolecules using classical mechanics remains a challenge (71,74), and although we feel the models used here represent the current state of the art and have predictive value, they may have limitations in their quantitative accuracy. In this regard, we do not discount the possibility that a Pb^2+^ ion may also make outer-sphere interactions with the 5′O leaving group, and in this binding mode, play a catalytic role as a general acid through a metal-bound water molecule as inferred crystallographically.

#### Role of Na^+^ (monovalent) metal ion

A striking feature of this work is the electrostatic engineering of the 8–17dz active site, and the identification of the supporting role of Na^+^ ions in catalysis. In the GB^−^ and AP simulations, a bridging Na^+^ ion plays an organizational role in bringing together the nucleophile and pro-*R*_P_ NPO and facilitating in-line fitness (α catalysis), while also positioning the nucleophile (tertiary γ catalysis) so as to form a productive hydrogen bond interaction with the G13:N1 heteroatom required for nucleophile activation. In this position, the Na^+^ ion also increases the acidity of the nucleophile to facilitate proton transfer to the general base (secondary γ catalysis), in a fashion similar to the role proposed for Lys41 in the classic RNA-cleavage mechanism of RNase A (55,75). Na^+^ ions also engage in a second form of secondary γ catalysis by p*K*_a_ tuning of G13:N1 through binding to the solvent-exposed Hoogsteen edge of the general base (G13:O6), similar to the recently proposed role of a Mg^2+^ ion in the hammerhead ribozyme (76–78). Finally, the Na^+^ ions play a minor supporting role to the Pb^2+^ ion in providing stabilization of the NPOs of the scissile phosphate and 5′O leaving group (β and δ catalysis).

#### Role of solvent molecules

In addition to interacting with key nucleic acid residues, the active site Pb^2+^ and Na^+^ ions coordinate water molecules as shown in Figure 7 and Supplementary Figure S7. Solvent analysis in the active site further suggests that there are significant bridging interactions that occur between G13:O6 and the G+1:pro-*S*_P_ positions mediated either by water molecules or Na^+^ ions (Supplementary Table S2) as depicted in Figure 5. These interactions are most prevalent in the active GB^−^ state, occurring with 70% occupancy, where they bring G13 and the scissile phosphate into close proximity and help to stabilize G13 in position to activate the nucleophile.

#### Simulations of the 8–17dz in the presence of Mg^2+^ require further study

As discussed previously, 8–17dz is most active in the presence of Pb^2+^ ions, but retains activity at reduced levels when Pb^2+^ ions are replaced with Mg^2+^ (or Zn^2+^) ions. Single-molecule FRET measurements suggest that 8–17dz undergoes further folding events into a more compact structure required for activity that is not observed or required in the presence of Pb^2+^ ions (26,27). Departing from the crystallographic data in the presence of a partially occupied Pb^2+^ ion, we sought to explore this metal-dependent folding in molecular simulations by replacing Pb^2+^ with Mg^2+^ and monitoring the end-to-end distance. We performed simulations of the GB^−^ and TS mimic states, departing from binding modes that involved alternatively (i) direct coordination to pro-*R*_P_, and (ii) direct coordination to both pro-*R*_P_ and G6:O6. The results unfortunately are not conclusive, since even though we observed a slight shortening in the end-to-end distance we were not able to clearly capture the full folding event (Supplementary Figure S8). We found two stable TS binding modes (Supplementary Figure S9), however further computational and/or experimental work is needed to refine and validate these models such that predictive insight can be gained into the specific binding mode and role of Mg^2+^ ion in folding and catalysis.

### Functional relation of 8–17 DNAzyme with the hammerhead and pistol ribozymes

The 8–17 DNAzyme is an RNA-cleaving enzyme that uses a divalent metal ion to assist in general acid (δ) catalysis, and shares some features with the hammerhead (HHr) and pistol (Psr) ribozymes (Supplementary Figure S10). In order to facilitate discussion of the divalent metal ion active site binding mode in 8–17dz, HHr and Psr, we designate this binding site as the ‘L-pocket’ that involves direct or indirect coordination of the metal ion with the pro-*R*_P_ NPO of the scissile phosphate and the Hoogsteen edge (O6/N7) of a guanine residue designated the ‘L-pocket G’; i.e. G6 in 8–17dz, G10.1 in HHr, and G33 in Psr.

In the extended (tertiary stabilized) hammerhead ribozyme crystal structures, either a Mn^2+^ ion (PDB ID: 2OEU (79)), or a Mg^2+^ ion (PDB ID: 5EAO (77)), is observed to directly coordinate the N7 of the L-pocket G, as well as the pro-*R*_P_ NPO of the scissile phosphate. Phosphorothioate-thiophilic metal ion rescue experiments (80,81), supported by molecular simulations (82,83), suggest that in the HHr active state the scissile phosphate acquires a functionally important inner-sphere coordination with a divalent metal ion that has yet to be observed crystallographically. Cd^2+^ rescue experiments on an extended HHr construct indicate that a catalytic metal ion may occupy this binding mode even in the ground state (84). In order to achieve this binding mode involving direct coordination to the pro-*R*_P_ NPO of the scissile phosphate, which is presumably required for formation of the active state, the direct coordination to N7 of the L-pocket G observed crystallographically must be disrupted. This is consistent with N7 deazaguanine substitution at the L-pocket G, which has only a ∼30-fold effect on activity in HHr. The generally supported role of the divalent ion in HHr, in addition to providing direct electrostatic stabilization of the transition state (primary β catalysis) is to increase the acidity of the 2′-OH of G8 (secondary δ catalysis), which can then engage in general acid (primary δ) catalysis. Alternative mechanisms for HHr have been suggested that involve a metal-bound water molecule that acts as a general acid (12,76,77,84) and it is possible that under different conditions multiple reactive channels are available (85).

In the Psr crystal structures (PDB ID: 5K7C (86), 5KTJ (87) and 6R47 (88)), a Mg^2+^ ion is observed to directly coordinate the N7 of the L-pocket G, as well as to indirectly coordinate the pro-*R*_P_ NPO of the scissile phosphate. Phosphorothioate-thiophilic metal ion rescue experiments have indicated thio substitution at the pro-*R*_P_ NPO of the scissile phosphate disrupts cleavage activity by 4.5 × 10^4^-fold in the presence of Mg^2+^, but in such a way that, unlike HHr, can only be partially (150-fold) rescued by more thiophilic Mn^2+^ ions (88). Also unlike in HHr, N7 deazaguanine substitution at the L-pocket G has a profound impact (300-fold (89) to 1.3 × 10^4^-fold (88) decrease) in activity. The role of the divalent ion in Psr is presumably to increase the acidity of a metal-bound water molecule (secondary δ catalysis) which can then engage in general acid (primary δ) catalysis.

In the 8–17dz crystal structure (PDB ID: 5XM8 (23)), a partially occupied Pb^2+^ ion is observed to directly coordinate the O6 of the L-pocket G, as well as to indirectly coordinate the pro-*R*_P_ NPO of the scissile phosphate. However, our simulations suggest that the preferred binding mode of this ion in the active state in solution is to involve indirect coordination to the Hoogsteen edge (O6/N7) of the L-pocket G, and direct coordination to the pro-*R*_P_ NPO of the scissile phosphate. In this sense, the predicted binding mode for 8–17dz is similar to that of HHr. Stereospecific thio substitution at the pro-*R*_P_ NPO of the scissile phosphate in 8–17dz can be used to selectively remove the *S*_P_ isomer in the presence of Mg^2+^, suggesting thio sensitivity at this position in the absence of thiophilic metal ions (67). More complete stereospecific thio/rescue effect measurements for 8–17dz are not available. Nonetheless, methylation at the O6 position of the L-pocket G in 8–17dz in the presence of Pb^2+^ has only a modest ∼28-fold effect on activity (23), similar to the analogous N7-deazaguanine mutational effect in HHr. This result is consistent with our simulation prediction that Pb^2+^ binding at the Hoogsteen edge of the L-pocket G observed crystallographically is important, but not critical for activity.

It is not clear the degree to which the binding mode and functional role of Pb^2+^ in 8–17dz may be transferable to more biologically friendly metal ions such as Mg^2+^. It is known that the inner-sphere binding of Mg^2+^ ions to the N7 position of guanines in RNA is quite rare (90), and although there is convincing evidence that this is likely the case for Psr, it seems less likely for 8–17dz and HHr. The Psr (88), HHr (91) and 8–17dz (92) all exhibit a linear-dependence of activity with p*K*_a_ of the metal ion. This result implicates the metal ion as playing an active role in the chemical steps of catalysis, but this feature is not able to discern the specific nature of that role. Quantum chemical simulations of the chemical steps of the reaction, together with stereospecific thio/rescue effect and metal ion titration experiments, would provide valuable insight to help resolve these important issues. In particular, it is possible that the predicted binding mode of the divalent metal ion at the pro-*R*_P_ NPO could be detected spectroscopically using Raman crystallography in a fashion similar to metal ion binding studies in the HDV ribozyme (93,94). Further, the catalytic role of the ion in the acid step of catalysis could be verified by measurement of acid rescue effects using an enhanced leaving group (e.g. 5′ thio substitution of the substrate), or alternatively linear free energy relations varying divalent metal ion p*K*_a_, as in recent studies of the pistol ribozyme (88), and interpreting results using multiscale QM/MM simulations (50). The role of G13 and its mechanistic implications in general or specific base catalysis could be probed by measurement of Brønsted coefficients using isofunctional guanine analogs that preserve the Watson–Crick face and exocyclic substituents, but provide systematic variation of the p*K*_a_ at the N1 position (95). The importance of Na^+^ ion interactions could be further examined by variation of monovalent ion concentration.

## CONCLUSION

We have performed simulations of the 8–17dz at four different stages along the reaction pathway in order to ascertain the mechanisms whereby the DNAzyme can support four fundamental catalytic strategies. Departing from recent crystallographic data, we identify the key requirements for formation of the active state in solution, and characterize the engineered architecture of an electrostatically strained active site capable of recruiting cationic charge to assist in catalysis. Simulations reveal the putative general base, G13, is positioned for general base catalysis, enabled by a bridging Na^+^ binding mode in the active site which also serves to support in-line fitness. The Pb^2+^ ion is observed to bind preferentially to the NPO of the scissile phosphate and provide direct electrostatic stabilization to the transition state and the accumulating charge of the O5′ leaving group, although a chemical mechanism whereby a metal-bound water molecule acts as a general acid is also plausible, it cannot be resolved by our current simulated predictions. A striking result of this work is the diverse role played by metal ions in the engineered 8–17dz that impact all four catalytic strategies, and share commonality with some features recently inferred for naturally occurring RNA enzymes such as the pistol and particularly the hammerhead ribozymes.

## Supplementary Material

gkz773_Supplemental_File
