# Supplementary material for: Dynamical ensemble of the active state and transition state mimic for the RNA-cleaving 8–17 DNAzyme in solution
Source: Nucleic Acids Res. 2019 Sep 12;47(19):10282–95. doi: 10.1093/nar/gkz773 (PMC6821293; doi:10.1093/nar/gkz773)
Supplement: gkz773_Supplemental_File [file gkz773_supplemental_file.pdf]

Supplementary Data for:

Dynamical ensemble of the active state and transition state mimic for the RNA-cleaving 8-17 DNAzyme in solution

Şölen Ekesan and Darrin M. York*

Laboratory for Biomolecular Simulation Research, Institute for Quantitative Biomedicine, and Department of Chemistry and Chemical Biology, Rutgers University, Piscataway, NJ 08854, USA

*To whom correspondence should be addressed. E-mail: Darrin.York@rutgers.edu

Supplementary Data

Fitness Indices for Catalytic Strategy Plots

Catalytic strategy analysis plots (Figures 4 and 5) are calculated and normalized in a strategy-specific manner. Alpha catalysis (in-line angle & distance) plot values are calculated as a 2D distance from the ideal in-line angle and distance point, normalized according to the cutoff values listed below. Beta catalysis ($M^{+}\cdots$NPO) plot values are calculated by linear combination of Pb^{2+} and Na^{+} scores weighed by the charge of the bound ion (i.e. roughly proportional to the neutralization/stabilization effect). Delta catalysis with $M^{+}\cdots O5'$ (i.e. Lewis acid) is calculated based on Pb^{2+} ion coordination only, whereas the delta catalysis with $Wat_{Pb}\cdots O5'$, Pb^{2+} coordinated water hydrogen bonding to the leaving group (i.e. general acid), is calculated by the hydrogen bond distance of the acidic water molecule directly coordinating Pb^{2+} to the $O5'$.

Intensity of the colors on the plots are based off of zero to one scoring of the property calculated as the distance of value from cutoff, normalized by the defined range between "best" value and cutoff; i.e., score = (value - cutoff) / (best - cutoff), where the "best" and "cutoff" are empirical fitness threshold values (when value=best, intensity score=1 and when value=cutoff, intensity score=0). Any value beyond the range is set to the value of the closest end point and having a corresponding intensity score at the boundary (i.e. 0 or 1). For in-line angle, the cutoff is set as 140° as is the standard, and the best value is chosen as 170° (in-line fitness values greater then 170° are shown with same intensity as 170°). For the remaining distance-related analysis, "best" and "cutoff" values are picked based on the radial distribution functions for each set of distance types as follows: in-line distance ($O2'\cdots P$: 3.1, 3.5 Å), sodium ion to any oxygen in first solvation shell ($Na^{+}\cdots O$: 2.0, 3.0 Å), lead ion to any oxygen within first and second solvation shells ($Pb^{2+}\cdots O$: 2.3, 5.5 Å), hydrogen bond distance from the hydrogen to the heavy atom ($H\cdots X$: 1.7, 2.2 Å).

Terminology and concepts regarding acid/base catalysis

We would like to clarify some terminology and concepts regarding acid/base catalysis, and relate them to the simulations in the current study. Often times in the ribozyme literature, the term "specific acid/base catalysis" is used to refer to catalysis involving OH^- and H_3O^+ species in solution, and "general acid/base catalysis" to refer to catalysis by other entities such as nucleobase residues. This utilization, however, is to some extent incomplete (1, 2). We wish to clarify that formally, specific acid/base catalysis involves complete, equilibrium proton transfer prior to the rate controlling step, whereas general acid/base catalysis involves proton transfer that occurs to some extent during formation of the rate-controlling transition state so as to affect kinetics. In this sense, to refer to specific residues as the "general base" or "general acid" without having a knowledge of mechanism where the roles of these residues have been confirmed is presumptuous.

In the present work, we examine models that mimic states along the presumed reaction pathway illustrated in Figure 1 of the main text using classical molecular dynamics. In doing so, these states are modeled as *stable states* in order to gain insight into how the DNAzyme environment can provide stabilization relevant for catalysis. It should be emphasized that in the catalytic mechanism itself, some of these states will be transient species along the reaction pathway, and not necessarily stable. For example, if the "activated precursor" (AP) state illustrated in the Figure 1b of the main text was a stable state where nucleophile activation was achieved in a proton transfer involving rapid equilibrium prior to formation of the rate controlling transition state, then this would imply a specific base catalysis mechanism. Further, note that our "transition state" (TS) mimic model is an idealized dianionic pentavalent phosphorane species, with no partial proton transfer from the general acid. In the actual catalytic mechanism, the reaction could conceivably proceed through a protonated phosphorane intermediate (although this is rare if there is a divalent metal ion coordinating the pro-R_P NPO), or more likely, a rate-controlling transition state that involves partial proton transfer to the leaving group.

In order to reconcile these issues, detailed study of the reaction pathway itself is needed using combined quantum mechanical/molecular mechanical simulations, and where possible validate experimentally through measurement of kinetic isotope effects (3, 4) and/or linear free energy relations (5, 6), as illustrated in recent work (7) However, in order to conduct such investigations, it is critical to first identify and characterize the active state in solution (8); i.e., the combination of active site configuration, protonation state and metal ion binding modes that are competent to catalyze the chemical steps of the reaction. For nucleic acid enzymes, this state is often not the most stable ground state, and can be very rarely sampled even under optimal conditions. The purpose of this work is to identify the dynamical ensemble of the active state of 8-17dz in solution, as well as make predictions about other relevant points along the reaction pathway based on models that mimic, but do not precisely represent, these states. In doing so, insight will be gained about key active site interactions along the reaction pathway that provide a basis for further experimental and theoretical studies that probe details of the catalytic mechanism.

Of tremendous importance is to ultimately experimentally verify the character of the predicted transition state according to a specific mechanism (4). Linear free energy relations (Brønsted "alpha" and "beta" correlations) provide insight into the sensitivity of the reaction to acid or base strength and reflect the extent of proton transfer in the rate controlling transition state (5, 9). In practice, Brønsted coefficients are measured by examination of the slope of plots of the intrinsic rate constant with the $\mathrm{p}K_a$ of the acid or base. However, in the case of enzyme catalysis, these measurements are often complicated by the fact that alterations to residues (e.g., nucleobases, amino acids or metal ions) that affect $\mathrm{p}K_a$ can also impact other aspects of catalysis. Very recently, LFERs for the divalent metal ion implicated in acid catalysis has been measured for the pistol ribozyme (10), and for the putative general base and acid residues in

the VS ribozyme[†] using precise "isofunctional" nucleobase chemical modifications. Perhaps the most sensitive probe of changes in the bonding pattern that occur in formation of the rate controlling transition state are kinetic isotope effects (3, 4, 11). Very recently, the Harris group has pioneered methods to accurately measure KIEs for 2′O-transphosphorylation reactions at the nucleophile, leaving group and non-bridge positions for the first time using native substrates (11, 12). These methods have been used extensive to study non-enzymatic RNA-cleavage via 2′O-transphosphorylation under both alkaline and acidic conditions (13, 14), as well as reactions catalyzed by RNase A (12) and Zn^{2+} ions (15). These measurements provide extremely valuable information about chemical bonding in the transition state that can be interpreted with computational methods to gain atomic-level insight (6, 12, 16, 17).

Overall Fold

The recent crystal structure of 8-17dz (18) provides key insight into the overall fold and architecture of the active site, and serves as a critical departure point for theoretical prediction of the dynamical ensemble in solution at different stages along the reaction pathway. The overall fold of 8-17dz is "V"-shaped, with P1 and P2 helical stems forming extended arms responsible for recognition of the substrate via canonical base pairing. The P1 and P2 helices make an angle of about 70 degrees, and come together at the catalytic core at the vertex of the "V" (Figure 2a). The catalytic core consists of 15 nucleotides that form a compact twisted pseudoknot containing two short helices (P3 and P4), oriented perpendicularly to one another. P3 contains three canonical Watson-Crick (WC) G=C/C=G base pairs with a semi-conserved C7 stacking the stem, whereas P4 contains the remaining four highly conserved nucleotides (19, 20) where G6 and C12 form a canonical WC C=G pair and A5 and G13 a non-canonical A□-▷G pair (21, 22). G13 and G6 are proposed to be the general base (18, 23) and metal ion binding site (18), respectively.

Residues T1 and A14 pack against P3 and P4, stacking with C10:G2 and A5:G13, respectively, and are positioned to orient the substrate nucleobases (G+1 and G-1) flanking the cleavage site. Note: we refer to the N-1 residue as G in the current work, despite that in some of the DNAzyme literature it is designated as rG (owing to the fact that it is the only RNA residue). T1 forms a T•G wobble pair with G+1, whereas A14 forms a non-canonical A□-▷G pair with G-1. These interactions cause a GG-kink (18) to occur at the substrate cleavage site, causing G-1 and G+1 to be splayed apart. This splaying of the substrate nucleobases flanking the scissile phosphate stabilizes the nucleophile to be in an in-line attack configuration, and is commonly seen in naturally occurring endonucleolytic ribozymes (24, 25).

Two additional key residues, T11 and C7, are located at the junctions between P3 and P4 helices and play a structural role to stabilize the pseudoknot. T11 is located at the J4/3 junction and stacks with G6:C12 of P4, whereas C7 is located at the J3/4 junction and stacks with C4:G8 of P3. Although T11 is not conserved, and C7 can tolerate mutation to a pyrimidine (19, 20), single nucleotide deletion of T11 and C7 leads to a 20-fold and 100-fold decrease in cleavage activity (18). The 8-17dz fold, facilitated by these interactions (illustrated in Figure S1), creates an architecture and electrostatic environment for the active site that enables key catalytic strategies to be brought to bear to enhance activity.

[†]Ganguly, A., Weissman, B. P., Giese, T. J., Li, N.-S., Hoshika, S., Rao, S., Benner, S. A., Piccirilli, J. A. and York, D. M. (2019) Theory and experiment converge to define the active site configuration and catalytic mechanism of the largest known nucleolytic ribozyme. *Nat. Chem.,* submitted for publication.

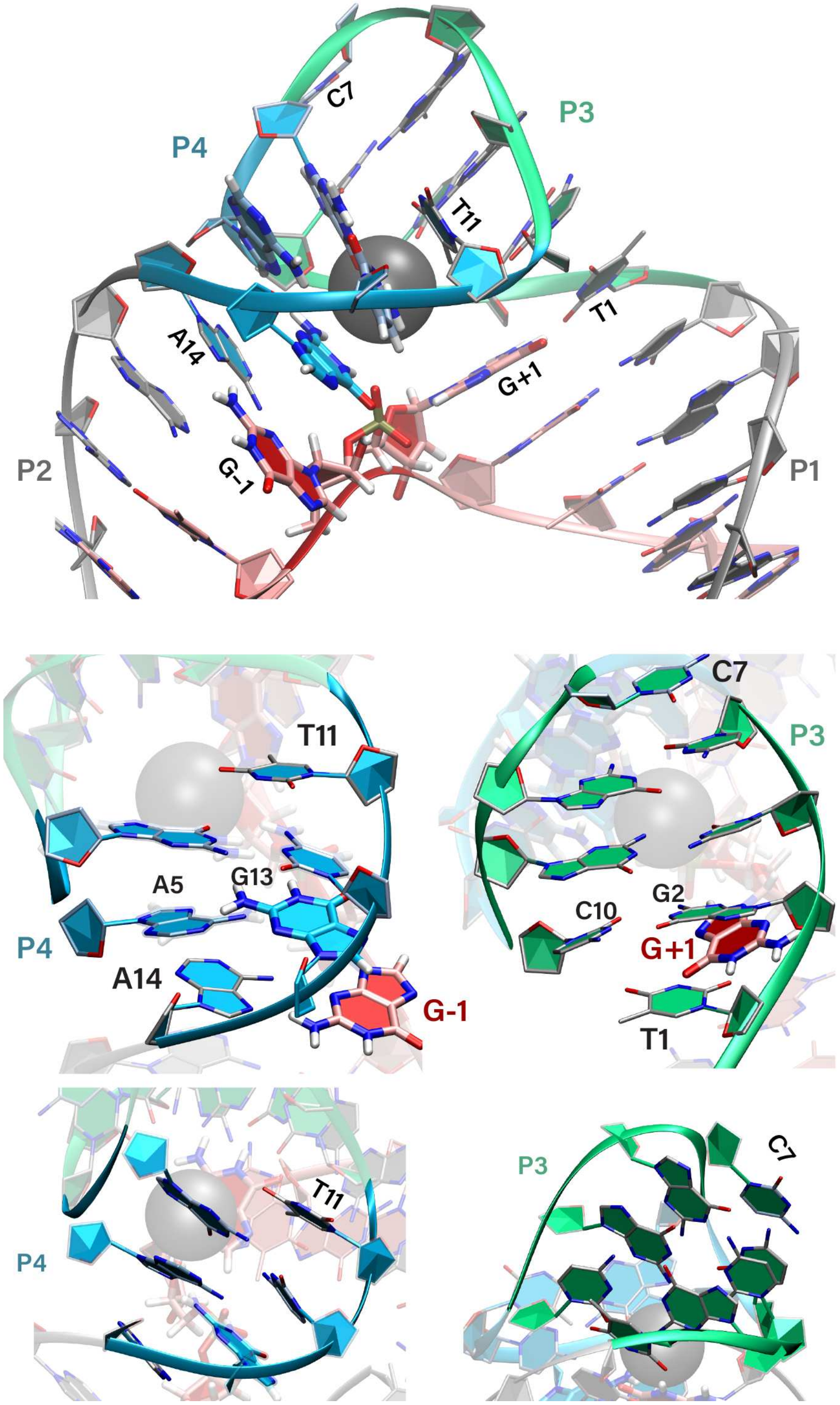

Figure S1. Crystal structure of the Pb^{2+} bound 8-17 DNAzyme (PDB ID: 5XM8 (18)) depicting catalytic core, from different view angles to emphasize P3 and P4 stems. The four stems of the enzyme are colored as: gray for substrate binding P1 and P2 stems, green for P3 stem containing canonical Watson-Crick (WC) pairing, and blue for the P4 stem with the catalytic conserved residues. The substrate strand is shown in pink (with the cleavage site highlighted in red).

Na^+ only simulation structure

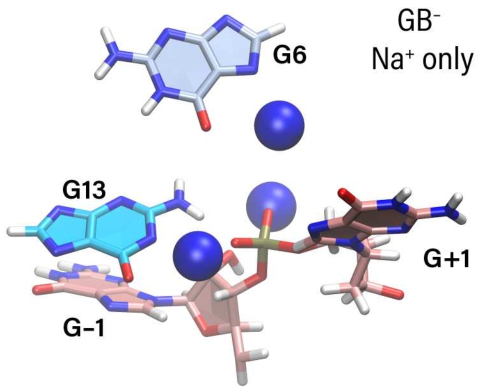

Figure S2. Solution structure of 8-17 DNAzyme from simulations of GB^- state without Pb^{2+} ("Na only"). Structure shown corresponds to average over trajectories. Cleavage site residues G-1 and G+1 on substrate strand are shown in pink, general base guanine G13 is in blue, and the conserved binding pocket residue G6 in light blue. All Na^+ ions within the binding pocket are shown (van der Waals representation, blue).

Clustering of GB^- frames

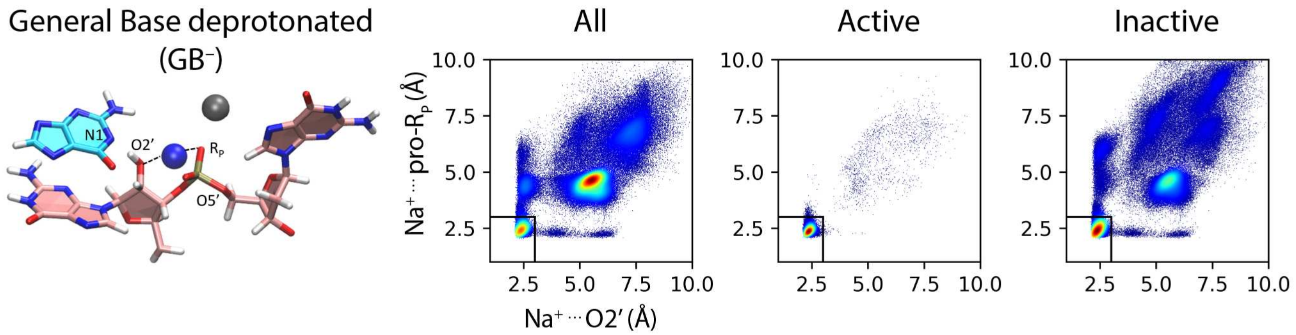

Figure S3. Na^+ ion population around 2′O nucleophile and pro-R_P oxygens in general base deprotonated (GB^-) state simulations with Pb^{2+} clustered as active and inactive. Densities are shown as increasing from blue to red.

Table S1. Summary of clustered frames. Number of frames belonging to each group (active vs inactive and Pb only vs Na* & Pb) are shown. Percent values in parentheses and brackets correspond to percentages along the row and column, respectively. In the simulations involving Pb^{2+}, the coordination environment of the Pb^{2+} ion can sometimes undergo small changes, but remains in the active site interacting with the scissile phosphate and leaving group.

	Active	Inactive	Total
Pb only	2004 (1.3%) [6.7%]	148735 (98.7%) [87.4%]	150739 (100.0%) [75.4%]
Na* & Pb	27726 (56.3%) [93.3%]	21535 (43.7%) [12.6%]	49261 (100.0%) [24.6%]
Total	29730 (14.9%) [100.0%]	170270 (85.1%) [100.0%]	200000 (100.0%) [100.0%]

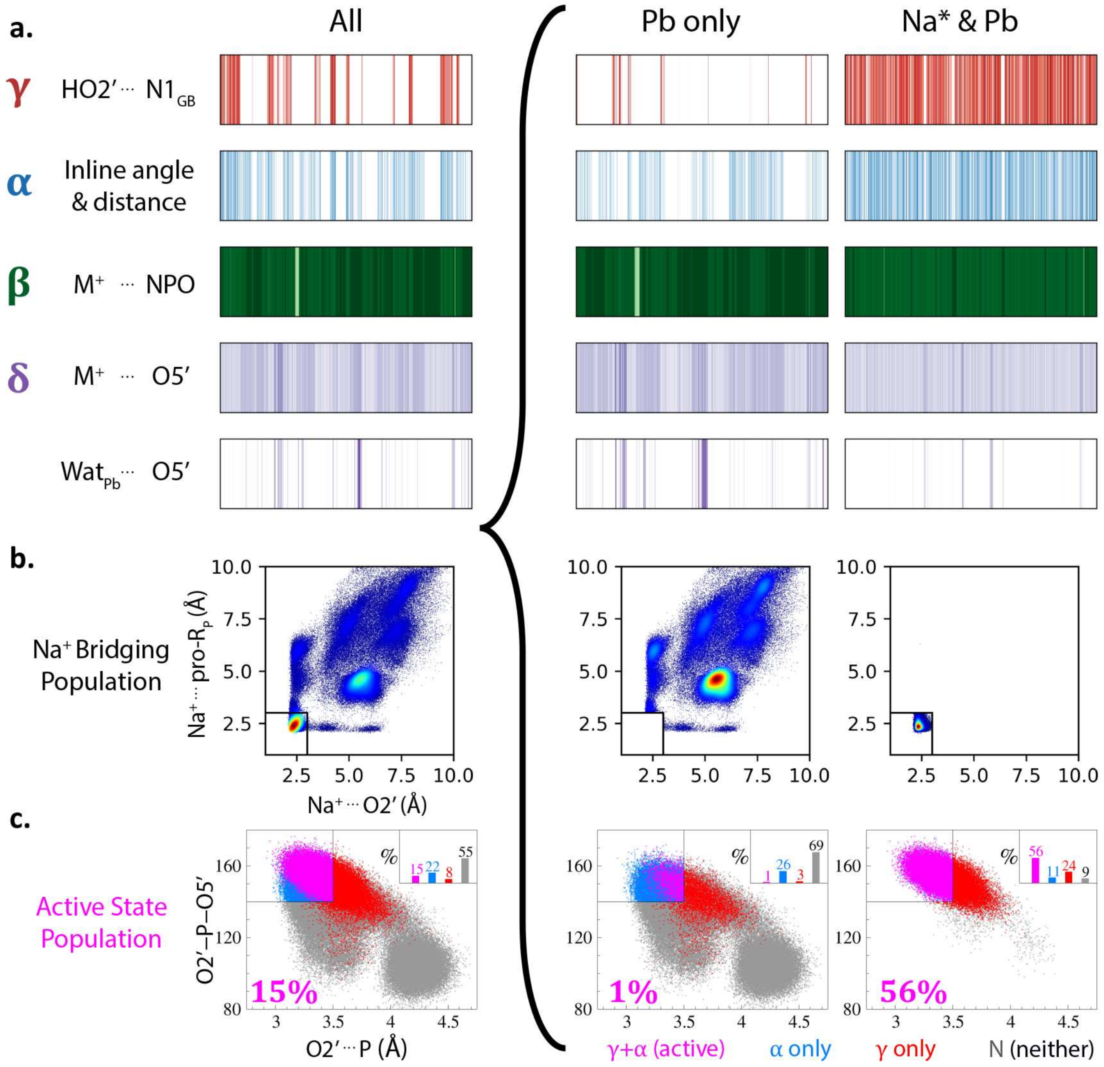

Figure S4. Clustering of general base deprotonated (GB^-) state trajectories based on presence of Na^+ ion in a bridging position between O2′ and pro-R_P. (a) Depiction of frames with active site positioned for nucleophile activation (γ, red), in-line attack (α, blue), charge stabilization of NPOs (β, green), and stabilization of the leaving group (δ, purple) along the trajectory. Frames matching the criteria for each strategy are shown in their corresponding colors, with darker tones indicating better catalytic fitness. Criteria for catalytic strategies are in-line attack (α: O2′···P ≤ 3.5 Å and ∠O2′-P-O5′ ≥140°), and nucleophile activation by general base (γ: HO2′···G13:N1 ≤ 3.5 Å). β criteria is based on direct coordination distance of a metal ion (Pb^{2+} and/or Na^+) to the NPOs. Two sets of analyses are shown for δ; metal ion coordination to the leaving group, and Pb^{2+} coordinated water hydrogen bonding to the leaving group. For metal ion coordination light and dark colors illustrate second and first solvation shell coordination, respectively. Criteria for Pb^{2+} coordinated water is based on the hydrogen bond distance to the leaving group O5′. (b) Na^+ ion population around 2′O nucleophile and pro-R_P oxygens with densities shown as increasing from blue to red. (c) Active state population shown as percentage of the overall frames in the category. Active state is defined as active site structure in position for both in-line attack (α) and nucleophile activation (γ). Points on the scatter plot are colored such that frames matching the criteria for only α or only γ are shown in blue and red, respectively. Frames that match criteria for both α and γ (i.e. active) are shown in magenta, and frames that do not match either criteria are shown in gray.

Solvent occupancy in bridging positions

Table S2. Percent occupancy of solvent molecules bridging between G13:O6 and G+1:pro-S_P. SS, AP and TS are percentages of 100k frames (i.e. 1 μs) and GB^- is percentage of 49k frames (i.e. 490 ns) from the active state cluster referred to as Na* & Pb.

%	SS	GB^-	AP	TS
1 water	19.8	66.1	29.1	57.9
2 waters	0.0	2.1	0.1	0.0
Na	3.1	2.1	2.1	0.3
Total	23.0	70.2	31.3	58.2

Restrained TS simulations

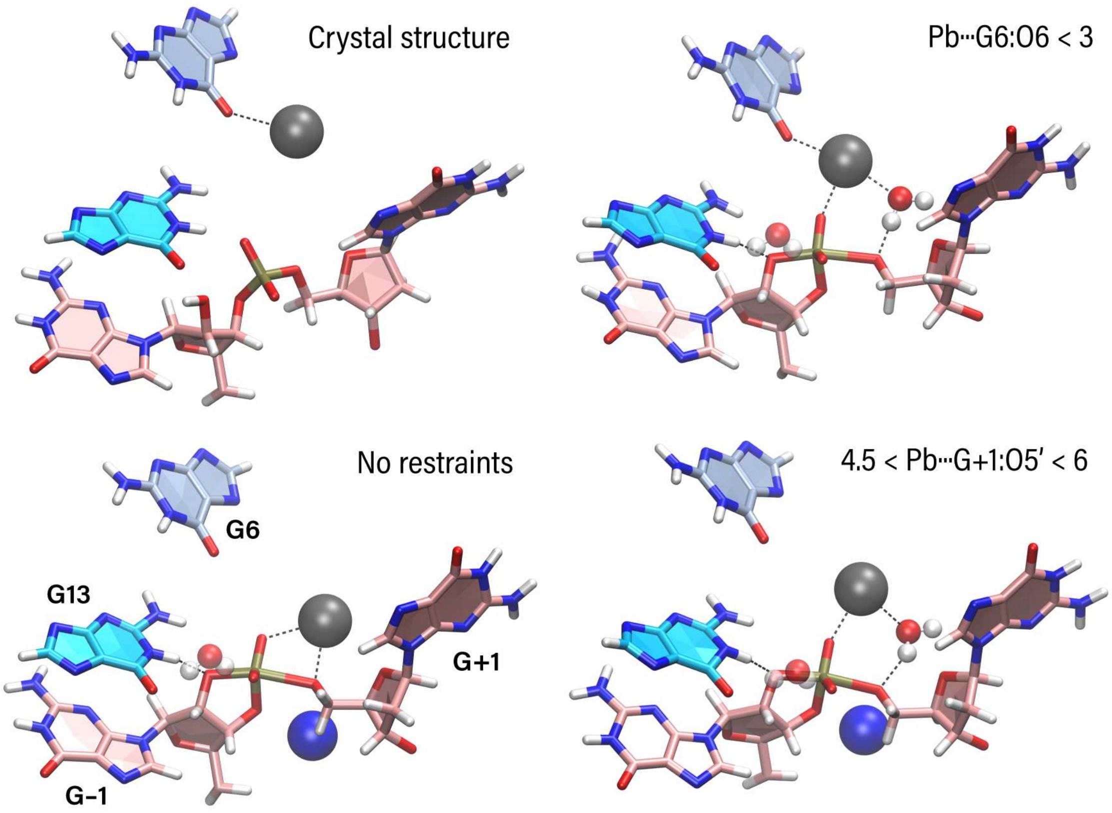

Figure S5. Solution structures of 8-17 DNAzyme from simulations of TS mimic states with and without restraints are shown along with the crystal structure. Cleavage site residues G-1 and G+1 on substrate strand are shown in pink, general base guanine G13 is in blue, and the conserved binding pocket residue G6 in light blue. Solvent molecules are shown when present in catalytically relevant positions. Metal ions are shown in van der Waals representation, Na^{+} in blue and Pb^{2+} in gray; and water molecules in CPK. Interactions within hydrogen bond or direct coordination distances are illustrated with black dashed lines.

Na^+ binding at G13

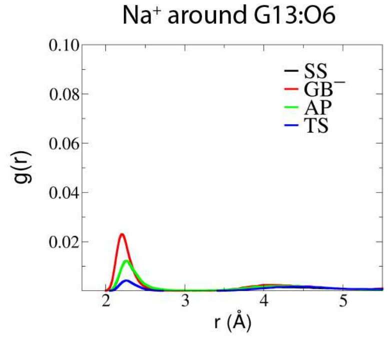

Figure S6. pK_a tuning of general base guanine by binding of metal ions at G13:O6. Radial distribution function, g(r), of Na^+ around G13:O6 obtained from solution simulations of standard state (SS, black), general base deprotonated state (GB^-, red), activated precursor state (AP, green), and transition state mimic (TS, blue) with Pb^{2+}. SS (black) line effectively invisible due to negligible peaks.

Interaction of water with ions in the active site

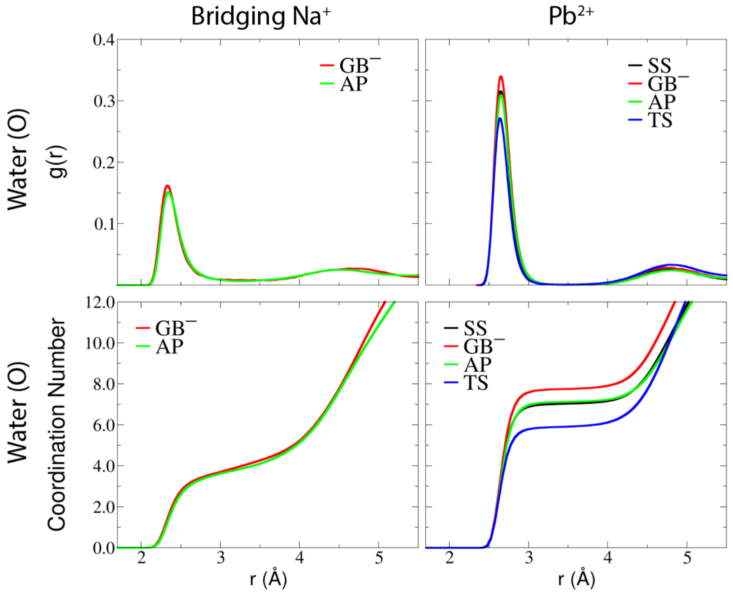

Figure S7. Solvent-ion interaction in the active site. Radial distribution function, g(r), and running coordination number (CN) of water molecules around active site bridging Na^+ and Pb^{2+} ions. Results with Pb^{2+} were obtained from solution simulations of standard state (SS, black), general base deprotonated state (GB^-, red), activated precursor state (AP, green), and transition state mimic (TS, blue) with Pb^{2+}. Results for bridging Na^+ were obtained only for the GB^- (red) derived from the cluster analysis in the active state (i.e., the "Na* & Pb" simulations), and AP (green). Average water coordination numbers for Na^+ are 3.9 in GB^- and 3.8 in AP. Average water coordination numbers for Pb^{2+} are 7.0 in SS, 7.7 in GB^-, 7.1 in AP and 5.8 in TS.

Preliminary Mg^{2+} Simulations

The dynamical ensemble of conformations exhibited by 8-17dz in solution is sensitive to both divalent (26, 27) and monovalent (28) ionic conditions. 8-17dz is most active in the presence of Pb^{2+} ions, but retains activity at reduced levels when Pb^{2+} ions are replaced with Mg^{2+} (or Zn^{2+}) ions. Single-molecule FRET measurements suggest that, in the presence of Mg^{2+} ions, 8-17dz further folds into a more compact structure required for activity that is not observed or required in the presence of Pb^{2+} ions (26, 27). Departing from the crystallographic data in the presence of a partially occupied Pb^{2+} ion, we sought to explore this metal-dependent folding in molecular simulations by replacing Pb^{2+} with Mg^{2+} and monitoring the end-to-end distance. We performed simulations of the GB^- and TS mimic states, departing from binding modes that involved alternatively 1) direct coordination to pro-R_P, and 2) direct coordination to both pro-R_P and G6:O6. The results are shown in Figures S8 and S9.

We ran these binding modes for the GB^- state in the presence of Mg^{2+}, however we could not obtain a stable active state as in the presence of Pb^{2+}. Nevertheless, since folding precedes activity we combined the trajectories (300ns), analyzed the end-to-end distances and compared to the values we get from Na^+ only and Pb^{2+} simulations. Figure S8 indicates there is a slight shift in the peak of the distribution of end-to-end distances for the simulation in the presence of Mg^{2+} relative to the distributions derived from the Pb^{2+} simulations. However, the distribution for the Mg^{2+} simulations is also more narrow, possibly as a result of less overall sampling and/or less dynamic binding modes of the smaller, harder Mg^{2+} ion that exhibits slower ligand exchange. Overall, the distributions are not reflective of the more substantial differences suggested by FRET measurements.

We similarly ran these binding modes for the TS state in the presence of Mg^{2+}, and obtained trajectories with binding modes stable for 100ns (Figure S9). Longer simulations and further analyses are required to test the stability and catalytic relevance of these binding modes.

Our results for Mg^{2+}-bound 8-17dz unfortunately are not conclusive, since we were not able to clearly capture the folding event on the time scale of the simulations, and did not observe formation of the active state at significant occupancy. It is likely that the time scales of the simulations (100 ns) is not sufficient to observe the Mg^{2+}-dependent folding event, and further sampling is required to make predictions that are consistent with the current body of available experimental data. Hence, further computational and/or experimental work is needed to gain insight into the specific binding mode and role of Mg^{2+} ion in folding and catalysis.

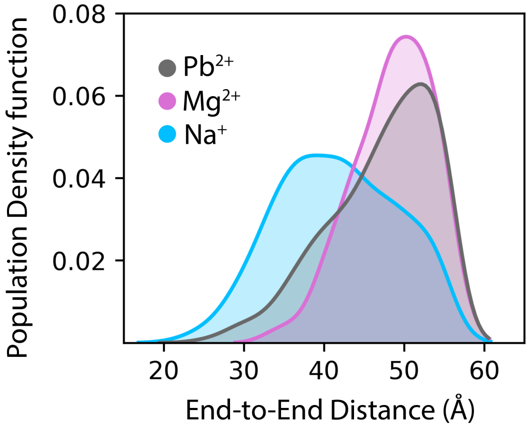

Figure S8. End-to-end distance distributions along trajectories for GB^- state simulations with Pb^{2+} (2 μs, gray), Mg^{2+} (300 ns, pink) and Na^+ (1 μs, blue). End-to-end distance calculated as the distance between the first phosphates in the 5′ end of each strand.

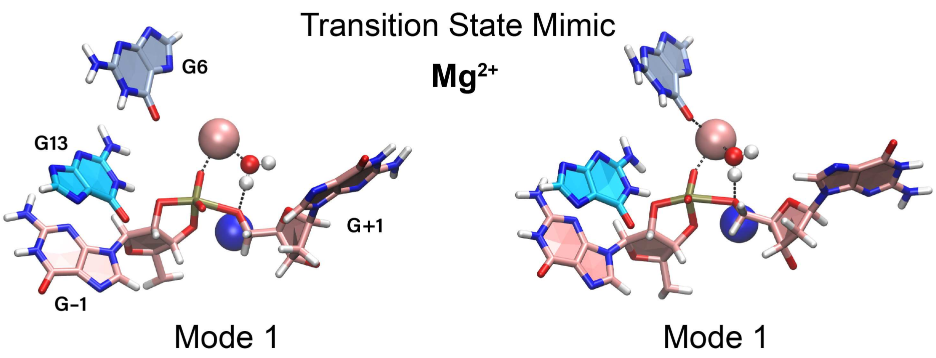

Figure S9. Two binding modes (Mode 1 and Mode 2) of Mg^{2+} in the transition state mimic stable for 100 ns. Cleavage site residues G-1 and G+1 on substrate strand are shown in pink, general base guanine G13 is in blue, and the conserved binding pocket residue G6 in light blue. Solvent molecules are shown when present in catalytically relevant positions. Metal ions are shown in van der Waals representation, Na^{+} in blue and Mg^{2+} in pink. Interactions within hydrogen bond or direct coordination distances are illustrated with black dashed lines.

Ribozyme Comparison

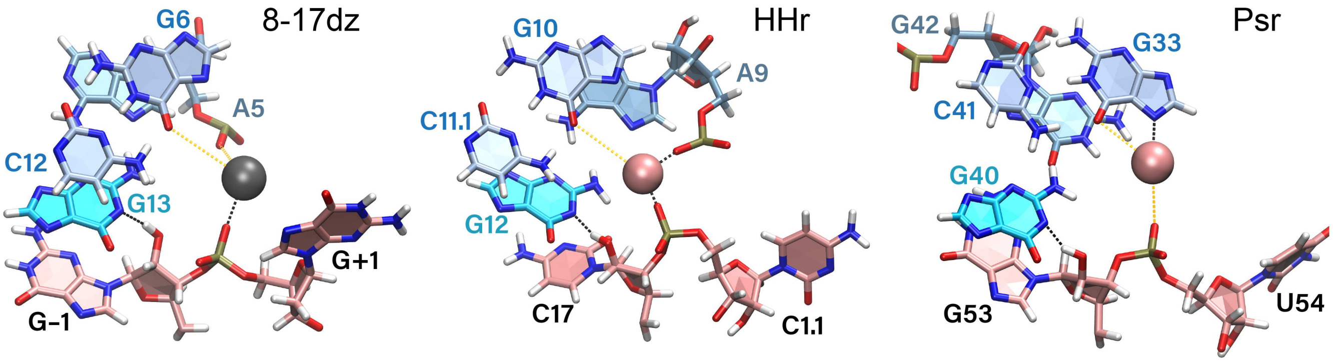

Figure S10. Active site comparison of 8-17dz with Hammerhead (HHr) and Pistol (Psr) ribozymes. HHr and Psr active states are results of MD simulations starting from PDB IDs 2OEU (29) and 5K7C (30), respectively. Cleavage site residues are shown in pink, general base guanine in bright blue, and the conserved active site residues in light blue. Labels are color grouped accordingly with the exception of general base anchoring residue (A/G) in slightly different tone as it is different among the systems compared. Metal ions are shown in van der Waals representation, Pb^{2+} in gray and Mg^{2+} in pink. Interactions within hydrogen bond or direct coordination distances are illustrated with black dashed lines, and indirect coordination with yellow dashed lines.

References

1. Jencks, W. P. (1969) Catalysis in Chemistry and Enzymology, McGraw-Hill, New York.

2. Jencks, W. P. (1972) General acid-base catalysis of complex reactions in water. *Chem. Rev.,* **72**, 705–718.

3. Hengge, A. C. (2002) Isotope effects in the study of phosphoryl and sulfuryl transfer reactions. *Acc. Chem. Res.,* **35**, 105–112.

4. Harris, M. E., Piccirilli, J. A. and York, D. M. (2015) Integration of kinetic isotope effect analyses to elucidate ribonuclease mechanism. *Biochim. Biophys. Acta,* **1854**, 1801–1808.

5. Jencks, W. P. (1985) A Primer for the Bema Hapothle. An Empirical Approach to the Characterization of Changing Transition-State Structures. *Chem. Rev.,* **85**(6), 511–527.

6. Chen, H., Giese, T. J., Huang, M., Wong, K.-Y., Harris, M. E. and York, D. M. (2014) Mechanistic Insights into RNA Transphosphorylation from Kinetic Isotope Effects and Linear Free Energy Relationships of Model Reactions. *Chem. Eur. J.,* **20**, 14336–14343.

7. Gaines, C. S., Giese, T. J. and York, D. M. (2019) Cleaning Up Mechanistic Debris Generated by Twister Ribozymes Using Computational RNA Enzymology. *ACS Catal.,* **9**(7), 5803–5815.

8. Gaines, C. S. and York, D. M. (2016) Ribozyme Catalysis with a Twist: Active State of the Twister Ribozyme in Solution Predicted from Molecular Simulation. *J. Am. Chem. Soc.,* **138**(9), 3058–3065.

9. Anslyn, E. V. and Dougherty, D. A. (2006) Modern Physical Organic Chemistry, University Science Books, Sausalito, CA.

10. Wilson, T. J., Liu, Y., Li, N. S., Dai, Q., Piccirilli, J. A. and Lilley, D. M. (2019) Comparison of the structures and mechanisms of the pistol and hammerhead ribozymes. *J. Am. Chem. Soc.,* **141**, 7865–7875.

11. Harris, M. E. and Cassano, A. G. (2008) Experimental analyses of the chemical dynamics of ribozyme catalysis. *Curr. Opin. Chem. Biol.,* **12**, 626–639.

12. Gu, H., Zhang, S., Wong, K.-Y., Radak, B. K., Dissanayake, T., Kellerman, D. L., Dai, Q., Miyagi, M., Anderson, V. E., York, D. M. et al. (2013) Experimental and computational analysis of the transition state for ribonuclease A-catalyzed RNA 2'-*O*-transphosphorylation. *Proc. Natl. Acad. Sci. USA,* **110**, 13002–13007.

13. Cassano, A. G., Anderson, V. E. and Harris, M. E. (2002) Evidence for Direct Attack by Hydroxide in Phosphodiester Hydrolysis. *J. Am. Chem. Soc.,* **124**, 10964–10965.

14. Harris, M. E., Dai, Q., Gu, H., Kellerman, D. L., Piccirilli, J. A. and Anderson, V. E. (2010) Kinetic isotope effects for RNA cleavage by $2'$-O-transphosphorylation: Nucleophilic activation by specific base. *J. Am. Chem. Soc.,* **132**, 11613–11621.

15. Zhang, S., Gu, H., Chen, H., Strong, E., Ollie, E. W., Kellerman, D., Liang, D., Miyagi, M., Anderson, V. E., Piccirilli, J. A. et al. (2016) Isotope effect analyses provide evidence for an altered transition state for RNA 2'-O-transphosphorylation catalyzed by Zn^{2+}. *Chem. Commun.,* **52**, 4462–4465.

16. Wong, K.-Y., Gu, H., Zhang, S., Piccirilli, J. A., Harris, M. E. and York, D. M. (2012) Characterization of the reaction path and transition states for RNA transphosphorylation models from theory and experiment. *Angew. Chem. Int. Ed.,* **51**, 647–651.

17. Chen, H., Piccirilli, J. A., Harris, M. E. and York, D. M. (2015) Effect of Zn^{2+} binding and enzyme active site on the transition state for RNA 2'-O-transphosphorylation interpreted through kinetic isotope effects. *Biochim. Biophys. Acta, Proteins Proteomics,* **1854**, 1795–1800.

18. Liu, H., Yu, X., Chen, Y., Zhang, J., Wu, B., Zheng, L., Haruehanroengra, P., Wang, R., Li, S., Lin, J. et al. (2017) Crystal Structure of an RNA-Cleaving DNAzyme. *Nat. Commun.,* **8**(1), 2006–2015.

19. Peracchi, A., Bonaccio, M. and Clerici, M. (2005) A mutational analysis of the 8–17 deoxyribozyme core. *J. Mol. Biol.,* **352**, 783–794.

20. Saran, R. and Liu, J. (2016) A comparison of two classic Pb^{2+}-dependent RNA-cleaving DNAzymes. *Inorg. Chem. Front.,* **3**, 494–501.

21. Leontis, N. B. and Westhof, E. (2001) Geometric nomenclature and classification of RNA base pairs. *RNA,* **7**, 499–512.

22. Leontis, N. B., Stombaugh, J. and Westhof, E. (2002) The non-watson-crick base pairs and their associated isostericity matrices. *Nucleic Acids Res.,* **30**, 3497–3531.

23. Cepeda-Plaza, M., McGhee, C. E. and Lu, Y. (2018) Evidence of a general acid–base catalysis mechanism in the 8–17 DNAzyme. *Biochemistry,* **57**, 1517–1522.

24. Bevilacqua, P. C., Harris, M. E., Piccirilli, J. A., Gaines, C., Ganguly, A., Kostenbader, K., Ekesan, Ş. and York, D. M. (2019) An Ontology for Facilitating Discussion of Catalytic Strategies of RNA-Cleaving Enzymes. *ACS Chem. Biol.,* **14**, 1068–1076.

25. Seith, D. D., Bingaman, J. L., Veenis, A. J., Button, A. C. and Bevilacqua, P. C. (2018) Elucidation of Catalytic Strategies of Small Nucleolytic Ribozymes from Comparative Analysis of Active Sites. *ACS Catal.,* **8**, 314–327.

26. Kim, H.-K., Rasnik, I., Liu, J., Ha, T. and Lu, Y. (2007) Dissecting metal ion–dependent folding and catalysis of a single DNAzyme. *Nat. Chem. Biol.,* **3**(12), 763.

27. Kim, H.-K., Liu, J., Li, J., Nagraj, N., Li, M., Pavot, C. M.-B. and Lu, Y. (2007) Metal-dependent global folding and activity of the 8-17 DNAzyme studied by fluorescence resonance energy transfer. *J. Am. Chem. Soc.,* **129**(21), 6896–6902.

28. Mazumdar, D., Nagraj, N., Kim, H.-K., Meng, X., Brown, A. K., Sun, Q., Li, W. and Lu, Y. (2009) Activity, folding and Z-DNA formation of the 8–17 DNAzyme in the presence of monovalent ions. *J. Am. Chem. Soc.,* **131**, 5506–5515.

29. Martick, M., Lee, T.-S., York, D. M. and Scott, W. G. (2008) Solvent structure and hammerhead ribozyme catalysis. *Chem. Biol.,* **15**, 332–342.

30. Ren, A., Vusurovic, N., Gebetsberger, J., Gao, P., Juen, M., Kreutz, C., Micura, R. and Patel, D. (2016) Pistol Ribozyme Adopts a Pseudoknot Fold Facilitating Site-specific In-line Cleavage. *Nat. Chem. Biol.,* **12**, 702–708.
